# Adipose single cell epigenome and transcriptome localize genetic risk for cardiometabolic disease and accelerated aging

**DOI:** 10.1038/s41467-026-72248-4

**Published:** 2026-04-20

**Authors:** Seung Hyuk T. Lee, Asha Kar, Kyla Z. Gelev, Zeyuan Johnson Chen, Sankha Subhra Das, Sini Heinonen, Tuure Saarinen, Maija Vaittinen, Dorota Kaminska, Hilkka Peltoniemi, Chongyuan Luo, Anne Juuti, Ville Männistö, Jussi Pihlajamäki, Minna U. Kaikkonen, Kirsi H. Pietiläinen, Brunilda Balliu, Päivi Pajukanta

**Affiliations:** 1 Department of Human Genetics, David Geffen School of Medicine at UCLA, Los Angeles, CA USA; 2 Bioinformatics Interdepartmental Program, UCLA, Los Angeles, CA USA; 3 Department of Computer Science, University of California, Los Angeles, CA USA; 4 Department of Computational Medicine, David Geffen School of Medicine at UCLA, Los Angeles, CA USA; 5 Department of Biotechnology, Sister Nivedita University, Kolkata, West Bengal India; 6 Obesity Research Unit, Research Program for Clinical and Molecular Metabolism, Faculty of Medicine, University of Helsinki, Helsinki, Finland; 7 Department of Abdominal Surgery, Abdominal Center, Helsinki University Hospital and University of Helsinki, Helsinki, Finland; 8 Institute of Public Health and Clinical Nutrition, University of Eastern Finland, Kuopio, Finland; 9 Department of Medicine, Division of Cardiology, UCLA, Los Angeles, CA USA; 10 Eira Hospital, Helsinki, Finland; 11 Institute of Clinical Medicine, School of Medicine, University of Eastern Finland, Kuopio, Finland; 12 Department of Medicine, Internal Medicine, Kuopio University Hospital, Kuopio, Finland; 13 Department of Medicine, Endocrinology and Clinical Nutrition, Kuopio University Hospital, Kuopio, Finland; 14 A. I. Virtanen Institute for Molecular Sciences, University of Eastern Finland, Kuopio, Finland; 15 Healthy Weight Hub, Abdominal Center, Helsinki University Hospital and University of Helsinki, Helsinki, Finland; 16 Department of Pathology and Laboratory Medicine, David Geffen School of Medicine at UCLA, Los Angeles, CA USA; 17 Department of Biostatistics, UCLA Fielding School of Public Health, Los Angeles, CA USA; 18 Institute for Precision Health, David Geffen School of Medicine at UCLA, Los Angeles, CA USA

**Keywords:** Obesity, Transcriptomics, Epigenomics, Type 2 diabetes

## Abstract

Obesity impairs subcutaneous adipose tissue function, which predisposes to chronic cardiometabolic comorbidities and accelerated biological aging. However, regulatory variants, their target genes and epigenomic landscape underlying this predisposition in each subcutaneous adipose tissue cell-type remain elusive. Our subcutaneous adipose tissue cell-type level *cis*-expression quantitative trait and colocalization analyses reveal *ci*s-expression quantitative trait locus variants, regulating 279 genes for 33 cardiometabolic disease and aging traits. Most of these genes are cell-type-specific (90%), led by adipocytes (55%), and missed in previous bulk tissue colocalization studies. Conducting subcutaneous adipose tissue cell-type level epigenome analysis, we discover that the vast majority (81%) of these colocalized cardiometabolic disease and aging risk variants map to the active chromatin compartments that comprise only 45% of the human genome, revealing three-dimensional epigenome in the center of cardiometabolic disease and aging risk. These findings uncover genetic and epigenomic regulation of genes underlying 33 cardiometabolic disease and aging traits in subcutaneous adipose tissue cell-types and offer critical insights into the principal role of three-dimensional chromatin in disease risk.

## Abstract (web summary)

Obesity predisposes to cardiometabolic comorbidities and accelerated biological aging. Here, the authors identify cell-type level regulatory variants and their epigenomic landscape underlying this predisposition in subcutaneous adipose tissue.

## Introduction

Obesity increases risk for cardiometabolic diseases (CMDs) and accelerates biological aging^1–3^; however, susceptibility to obesity-associated comorbidities varies widely across individuals^4,5^. Identifying disease susceptibility loci for obesity-associated comorbidities could provide insights into this variability. Expression quantitative trait loci (eQTLs) have been widely used along with disease-associated variants from genome-wide association studies (GWASs) to discover genes and regulatory variants underlying disease mechanism in relevant tissues^6,7^. Subcutaneous adipose tissue (SAT), a primarily fat storing tissue and an endocrine organ^8,9^, has been linked to early signs of CMDs and accelerated biological aging^4,10^. Previous large SAT bulk eQTL studies have identified regulatory variants for CMD-associated GWAS variants through colocalization analyses^11–13^. However, these prior bulk tissue studies are confounded by cellular heterogeneity, limiting resolution of gene-trait associations, masking causal mechanisms, and leaving the SAT cell-types underlying disease mechanisms unknown. Furthermore, the previous SAT bulk tissue colocalization studies^11–13^ are also largely limited to relatively healthy individuals, which may limit the detection of variants in cell-types responding to obesity and inflammation.

Single cell and single nucleus RNA-sequencing (snRNA-seq) have been used on several tissues such as brain and lung for cell-type level mapping of eQTLs and to discover additional regulatory variants previously undetected in bulk RNA-seq of corresponding tissues^14–17^. Single cell transcriptomic studies further revealed that many disease-associated regulatory variants are in fact cell-type-specific and context-dependent^7,18^, emphasizing the importance of investigating cell-type level eQTLs that have not been explored in earlier SAT bulk tissue studies^11–13^. At the same time, recent single cell epigenomic atlases profiling human cell-type level DNA methylation and three-dimensional (3D) genome organization revealed cell-type-specific epigenomic regulatory regions^19,20^; however, these epigenomic resources have not been analyzed together with cell-type level eQTLs. Together, these gaps motivate a SAT, obesity-context, cell-type-resolved eQTL map linked to 3D chromatin to localize disease mechanisms.

In this study, we combined genotype data with snRNA-seq datasets of SAT biopsies from individuals with obesity to map cell-type level *cis*-eQTLs across five SAT cell-types and then leveraged cell-type level SAT epigenomics data to localize regulatory mechanisms. We discover colocalized eQTL and GWAS variants for 33 CMD and biological aging traits, many of which are cell-type-specific and have not been found in the recent largest SAT bulk meta-analysis study^13^. By integrating SAT cell-type level single nucleus methyl-3C sequencing (snm3C-seq) data^20^, we delineate distinct epigenomic regulatory architecture for the colocalized variants linked to CMD and accelerated biological aging. Finally, we utilize colocalized variants to predict type 2 diabetes (T2D) diagnosis and test their associations with obesity, demonstrating how cell-type level results prioritize regulatory variants for T2D risk prediction.

## Results

### Subcutaneous adipose tissue bariatric surgery cohorts

**Fig. 1:**
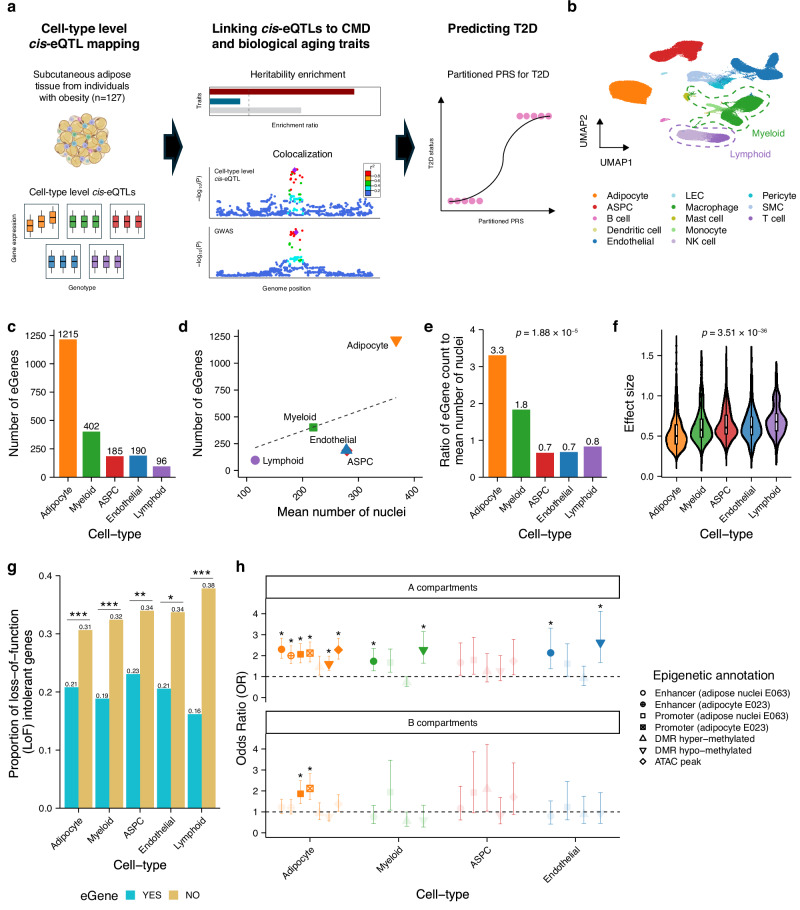
*Cis*-eQTL mapping at cell-type resolution discovers heterogeneity in genetic regulation across SAT cell-types. **a** Study overview. Adipose tissue icon created in BioRender. Lee, S.H.T. (2026) https://BioRender.com/klfxupf. **b** Uniform Manifold Approximation and Projection (UMAP) visualization of 124,664 nuclei (*n* = 127 SAT biopsies). Colors represent SAT cell-types and dashed lines the cell-types with a common lineage combined for *cis*-eQTL mapping. **c** Bar plots showing the number of genes with a significant *cis*-eQTL in each cell-type. **d** Scatter plot showing for each cell-type the mean number of nuclei per individual and eGene count, with the dashed line indicating the linear fit. **e** Ratio of eGenes to the mean number of nuclei per individual for each cell-type. *P*-values represent one-sided z-score test for the probability of the adipocyte ratio being a significant outlier. **f** Violin plots with inner boxplots showing effect sizes of cell-type level lead *cis*-eQTLs. Center lines of boxes indicate the median magnitude of variant effect sizes of the lead *cis*-eQTLs, boxes range 25th–75th percentiles, whiskers show 1.5× interquartile range (IQR), and black dots represent outliers. The median magnitude of the variant effect size was compared across cell-types using two-sided Kruskal-Wallis test; *n* = 1215, 402, 185, 190, and 96 for adipocyte, myeloid, ASPC, endothelial, and lymphoid cells. **g** Proportion of cell-type expressed LoF intolerant genes (pLI ≥ 0.9). Colors represent eGene status. Asterisks indicate significant differences in proportions by eGene status (*p*-values from Chi-square tests after Bonferroni correction for cell-type number: *, *p*.adj <0.05; **, *p*.adj <0.01; ***, *p*.adj <0.001) (exact *p*-values shown in Supplementary Data 6). **h** Lead *cis*-eQTLs in the cell-type level active A vs inactive B chromatin compartments enriched in *cis*-regulatory elements and epigenetic features. Dots represent odds ratios (ORs), error bars 95% confidence intervals of ORs, dashed lines OR = 1, colors cell-type, shapes epigenetic annotation, and asterisks and opacity significant enrichments using two-sided test from GARFIELD^87^ after Bonferroni correction (exact *p*-values and *n* values of 40 tests shown in Supplementary Data 10). ASPC adipose stem and progenitor cells, eQTL expression quantitative trait locus, LEC lymphatic endothelial cells, NK natural killer cells, SMC smooth muscle cells.

To map cell-type level *cis*-eQTLs in individuals with extreme obesity, we combined two single nucleus RNA-sequencing (snRNA-seq) datasets, comprising 127 subcutaneous adipose tissue (SAT) biopsies from two Finnish bariatric surgery cohorts: Kuopio Obesity Surgery Study (KOBS; *n* = 59) and Roux-en-Y versus one-anastomosis gastric bypass study (RYSA; *n* = 68)^21^ (Fig. 1a). All biopsies were collected at the time of bariatric surgery from individuals with a mean BMI of 42.1 kg/m^2^ (standard deviation, SD = 5.0), of whom 33% have T2D (for clinical characteristics, see Supplementary Data 1). We performed data quality control (QC) and cell-type annotation for each dataset by applying the same pipeline and then integrated the two datasets, resulting in 124,664 high-quality nuclei, representing 13 SAT cell-types (Fig. 1b, “Methods”, and Supplementary Methods). For conceptual unity, we use the term “single cell” to refer to the data generated from single nucleus assays hereinafter.

### Discovery and characterization of cell-type level *cis*-eQTLs

We performed cell-type level *cis*-eQTL mapping using pseudobulk mean expression per donor in each cell-type (“Methods”). To increase our power to map *cis*-eQTLs, we focused on the five most prevalent cell-types, including adipocytes, adipose stem and progenitor cells (ASPCs), endothelial cells, lymphoid cells (natural killer and T cells combined), and myeloid cells (macrophages and monocytes combined). We identified 1684 genes with at least one *cis*-eQTL variant (i.e., eGenes) across cell-types, with the greatest number in adipocytes (*n* = 1215) (Fig. 1c and Supplementary Data 2). By employing a stepwise regression procedure (“Methods”), we also identified 3–112 conditionally independent secondary signals across the five cell-types as well as 10 and one tertiary signals in adipocytes and myeloid cells, respectively (Supplementary Data 3). Overall, the number of eGenes for a cell-type increased with both the mean number of nuclei and number of expressed genes (Fig. 1d, e and Supplementary Fig. 1a, b). However, we still identified significantly more eGenes in adipocytes than in other cell-types after accounting for the differences in the nuclei amount using the modified z-score method (*z* = 4.12; *p* = 1.88 × 10^−5^). To further confirm this finding, we performed a down-sampling analysis, in which the *cis*-eQTL mapping was repeated for adipocytes after randomly subsampling the nuclei to match the counts of endothelial and lymphoid cells for each individual (“Methods”). We discovered 808 and 500 eGenes in adipocytes when down-sampling to the number of endothelial and lymphoid cells, respectively. Thus, down-sampling the adipocytes still resulted in larger numbers of adipocyte eGenes than the 190 endothelial and 96 lymphoid cell eGenes we had identified. Lastly, the median magnitude of lead *cis*-eQTL effect size was significantly different across the cell-types, with adipocytes harboring more eQTLs with smaller effect sizes (Fig. 1f).

To evaluate the robustness of our *cis*-eQTL results, we first assessed replication of the primary lead *cis*-eQTL variants for each cell-type among bulk SAT *cis*-eQTLs from AdipoExpress^13^. Focusing on the lead gene-variant pairs tested in both studies, 54–78% of our cell-type level *cis*-eQTLs were also *cis*-eQTLs in the SAT bulk meta-analysis (*p* < 1 × 10^−6^) and 46–73% replicated with concordant effect direction (Supplementary Data 4). The replication rate was the lowest (46%) for the least abundant cell-type, lymphoid cells. However, adipocytes did not have the highest replication rate despite being the most abundant cell-type. The low sharing of eQTLs, together with more eGenes detected in adipocytes, may partly reflect altered adipocyte regulations under obesity, in line with the previous studies showing that obesity is linked to adipocyte heterogeneity^22–25^.

To further validate our *cis*-eQTL results, we also performed cell-type level *cis*-eQTL mapping using a single cell eQTL tool, SAIGE-QTL^26^ (“Methods”). We discovered 1340 eGenes in adipocytes, 295 in ASPCs, 289 in endothelial cells, 133 in lymphoid cells, and 560 in myeloid cells, respectively (Supplementary Data 5). These eGenes include 74–78% of the eGenes per cell-type that were discovered with pseudobulk mean expression using tensorQTL^27^, demonstrating a high degree of concordance between the two eQTL approaches. This result suggests that the identified cell-type level *cis*-eQTLs are robust and reproducible across different methodological frameworks.

As loss-of-function (LoF) intolerant genes have previously been shown to exhibit less bulk tissue eQTLs^28^, we asked whether this depletion also holds at the cell-type level in SAT. We compared the proportion of LoF intolerant genes between the cell-type expressed genes with and without a significant cell-type level *cis*-eQTL variant. Among the eGenes with probability of LoF intolerance (pLI) estimates available, 11–186 (16–23%) were LoF intolerant (pLI ≥ 0.9) in the different SAT cell-types (Fig. 1g and Supplementary Data 6). In contrast, cell-type expressed genes without a significant *cis*-eQTL variant had significantly higher proportions of LoF intolerant genes, accounting for 31–38% (Fig. 1g and Supplementary Data 6). By conducting a gene set overrepresentation analysis of Gene Ontology (GO) biological process, Reactome pathways, and WikiPathways, we found that the LoF intolerant eGenes were largely enriched for broad regulation of biological, cellular, and metabolic processes in all cell-types except lymphoid cells (Supplementary Data 7). In adipocytes, LoF intolerant eGenes were also enriched for GO biological processes that are essential for adipocyte functions, including fat cell differentiation and cellular response to lipid, as well as other functional pathways (Supplementary Data 7). These pathways include mitogen-activated protein kinase (MAPK) family signaling cascades and deubiquitination, well-established regulators of adipocyte differentiation^29,30^. Another example is the Human Thyroid Stimulating Hormone (TSH) signaling pathway, which is involved in regulation of lipolysis^31^. Thus, genetic regulation of the genes in these pathways may modify adipocyte differentiation and lipolysis, contributing to adipose tissue dysfunction and increased risk for obesity and metabolic complications. Lastly, we observed a significantly lower (adjusted *p* = 2.68 × 10^−2^) median magnitude of the adipocyte *cis*-eQTL effect size for the LoF intolerant than tolerant (pLI ≤ 0.1) eGenes (Supplementary Fig. 1c). These results demonstrate constraints on cell-type level *cis*-regulation of the LoF intolerant genes that likely require maintained expression for essential biological functions.

To examine *cis*-regulatory elements and epigenomic features that may underlie cell-type level *cis*-regulation of gene expression at the cell-type resolution, we assessed enrichment of primary lead *cis*-eQTL variants using (i) promoters and enhancers from the Roadmap Epigenomics human adipose nuclei sample E063^32,33^ and adipocyte sample E023^32,33^, (ii) open chromatin peaks from cultured human preadipocyte (i.e., ASPC) and differentiated adipocyte ATAC-seq^34^ data, and (iii) differentially methylated regions (DMRs; hyper- and hypo-methylated) from our SAT cell-type level single nucleus methyl-3C sequencing (snm3C-seq) data^20^ (“Methods”). Lead *cis*-eQTL variants were significantly enriched in promoters for adipocytes (odds ratio, OR = 2.0 and 2.1 using adipose nuclei E063 and adipocyte E023, respectively) and myeloid cells (OR = 1.8), enhancers for adipocytes (OR = 1.9 and 1.7 using adipose nuclei E063 and adipocyte E023, respectively), hypo-methylated regions for endothelial (OR = 2.2) and myeloid (OR = 1.9) cells, and ATAC peaks for adipocytes (OR = 2.0) (Supplementary Fig. 1d and Supplementary Data 8). Consistent with the greatest number of eGenes detected, adipocytes showed strong enrichments in a broad range of *cis*-regulatory elements and epigenomic features.

Spatial organization of chromatin structure, such as active A and inactive B compartments, is involved in gene regulation^35–37^. Thus, we further investigated whether three-dimensional (3D) chromatin compartments in different SAT cell-types may contribute to the regulation of *cis*-eQTLs. Accordingly, we leveraged cell-type level chromatin compartment annotations from our SAT snm3C-seq data^20^ for four available cell-types (adipocytes, ASPCs, endothelial and myeloid cells). Among the identified eGenes with chromatin compartment annotations available, 65–79% resided in the A compartments of the cell-type (Supplementary Fig. 1e). Notably, myeloid cell eGenes in A compartments were significantly enriched for immune-related pathways (Supplementary Data 9), which may reflect increased activation in genetic regulation of gene expression in myeloid cells in response to obesity induced low-grade inflammation. The eGenes residing within the other cell-type level A compartments did not show functional enrichments.

Stratifying epigenomic enrichments of lead *cis*-eQTL variants by chromatin compartments revealed a clear A compartment bias. *Cis*-eQTL variants in A compartments were enriched for enhancers and hypo-methylated regions in endothelial (OR = 2.1 and 2.6) and myeloid (OR = 1.7 and 2.3) cells. Adipocyte *cis*-eQTL variants in A compartments were also enriched for enhancers (OR = 2.3 and 2.0 using adipose nuclei E063 and adipocyte E023, respectively) and hypo-methylated regions (OR = 1.6), in addition to promoters (OR = 2.1 using both adipose nuclei E063 and adipocyte E023) and ATAC peaks (OR = 2.3) (Fig. 1h and Supplementary Data 10). By contrast, in B compartments, the only significant enrichment was for promoters in adipocytes (OR = 1.9 and 2.1 using adipose nuclei E063 and adipocyte E023, respectively) (Fig. 1h and Supplementary Data 10). Thus, lead *cis*-eQTL variants in cell-type level A compartments preferentially localized to *cis*-regulatory elements and epigenomic features, in line with the higher proportion of eGenes in A compartments.

### Most *cis*-eQTLs are observed in adipocytes

**Fig. 2:**
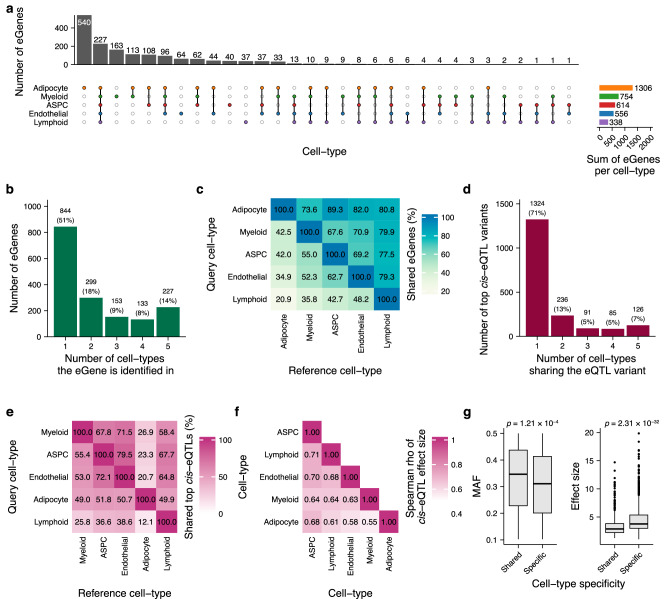
Adipocytes show the highest cell-type-specificity of *cis*-eQTLs in SAT. **a** Upset plot showing the number of cell-type-shared and cell-type-specific eGenes across combinations of cell-types. The horizontal bar plot (right panel) displays the total number of eGenes identified in each cell-type using mashr^38^. **b** Histogram showing cell-type sharing and specificity of the eGenes. Percentage of eGenes found in the different number of cell-types out of the total number of eGenes are shown in parentheses. **c** Heatmap showing percentage of eGenes overlapping in each pairwise combination of cell-types. Cell-types are ordered based on hierarchical clustering of the query cell-types. **d** Histogram showing cell-type sharing and specificity of the top independent lead *cis*-eQTL variants. Percentage of top *cis*-eQTL variants found in the different number of cell-types out of the total number of top *cis*-eQTL variants are shown in parentheses. **e** Heatmap showing percentage of top *cis*-eQTL variants overlapping in each pairwise combination of cell-types. Cell-types are ordered based on hierarchical clustering of the query cell-types. **f** Pairwise Spearman correlation for effect sizes of the top *cis*-eQTL variants. Cell-types are ordered based on hierarchical clustering. **g** Box plots comparing the median minor allele frequency (MAF) (left panel) and median magnitude of effect sizes (right panel) between the cell-type-shared (*n* = 538) and -specific (*n* = 1324) top *cis*-eQTL variants. Center lines of the boxes indicate the median MAF and magnitude of effect sizes for cell-type-shared and cell-type-specific *cis*-eQTL variants. Boxes range from the 25th–75th percentiles, whiskers show 1.5× the interquartile range (IQR), and black dots represent outliers. Two-sided Wilcoxon rank sum tests were performed to evaluate the significance of the differences in the median MAF and magnitude of effect sizes. ASPC adipose stem and progenitor cells, eQTL expression quantitative trait loci.

Sharing of *cis*-eQTL effects between heterogeneous SAT cell-types is not well understood. To this end, we examined cell-type sharing of *cis*-eQTL variants and their eGenes using mashr^38^. Overall, the greatest number of eGenes was discovered only in one cell-type, with adipocytes harboring 540 of 844 (64%) cell-type-specific eGenes (Fig. 2a, b). These 540 eGenes constitute 41% of 1306 adipocyte eGenes (local false sign rate, LFSR < 0.05). In comparison, 6.5–22% of the eGenes were cell-type-specific in other cell-types, which may reflect both greater power to detect *cis*-eQTLs and obesity-induced regulatory changes in adipocytes. Among the cell-type-shared eGenes, most were broadly shared across all five cell-types (*n* = 227 eGenes). The next most common pattern involved sharing between adipocytes and myeloid cells (*n* = 113 eGenes), consistent with the greater number of eGenes in these cell-types. However, pairwise eGene sharing was highest between adipocytes and ASPCs, the two biologically similar cell-types, with 89.3% of ASPC eGenes also present in adipocytes (Fig. 2c).

Next, we analyzed sharing of top *cis*-eQTL variants after LD pruning to remove redundant signals across cell-types. We defined top *cis*-eQTL variants as cell-type-shared if they had the same direction of effect and effect size within a factor of two when compared to the cell-type with the greatest effect size. As with eGenes, most top *cis*-eQTL variants were specific to one cell-type, comprising 71% (1324 out of 1862) of the total top *cis*-eQTL variants (Fig. 2d). In comparison, only 7% (126 out of 1862) were shared across all five cell-types (Fig. 2d). Adipocytes contributed the most cell-type-specific top *cis*-eQTL variants, followed by myeloid cells. Pairwise cell-type sharing of top *cis*-eQTL variants was highest between endothelial cells and ASPCs, where 79.5% of endothelial cell *cis*-eQTL variants were shared with ASPCs (Fig. 2e). Interestingly, only 51.8% of ASPC *cis*-eQTL variants were shared with adipocytes, in contrast to the 89.3% of the ASPC eGenes shared with adipocytes. These results suggest that while the genes under genetic regulation in these two biologically relevant cell-types overlap, the direction and magnitude of genetic effects are more cell-type-specific. Overall, *cis*-eQTL effect sizes were correlated across cell-type-shared variants, with a median Spearman rho of 0.64 (Fig. 2f). Finally, compared with cell-type-shared variants, cell-type-specific top *cis*-eQTL variants tended to have lower minor allele frequencies (MAFs) and larger absolute effect sizes (Fig. 2g), which may suggest that they regulate genes with essential cell-type-specific biological functions^7^.

To investigate whether cell-type-specific *cis*-eQTLs are also tissue-specific, we compared the replication of cell-type-specific top *cis*-eQTL variants (*n* = 1324) to bulk SAT and visceral adipose tissue (VAT) *cis*-eQTL results from GTEx^39^. Focusing on our 1096 SAT cell-type-specific variants that were tested in both SAT and VAT bulk tissue datasets, 329 (30%) and 250 (23%) of them were bulk SAT and VAT *cis*-eQTLs (*p* < 1 × 10^−6^), respectively, with a concordant direction of effect (Supplementary Data 11). This significantly greater number of SAT cell-type-specific *cis*-eQTLs discovered in the SAT than VAT bulk tissue *cis*-eQTL analysis of GTEx (adjusted *p* = 1.11 × 10^−14^ by one-sided McNemar’s test) supports the conclusion that the SAT cell-type-specific eQTLs are in part tissue-specific.

Next, we assessed tissue-specificity of the cell-type level lead *cis*-eQTL variants in each of the five SAT cell-types. Of our variants that were also tested in both SAT and VAT bulk tissue *cis*-eQTL analysis of GTEx, we observed that 37% vs 30% in adipocytes, 52% vs 41% in ASPCs, 56% vs 48% in endothelial cells, 31% vs 29% in lymphoid cells, and 43% vs 37% in myeloid cells were also *cis*-eQTLs (*p* < 1 × 10^−6^) in the SAT vs VAT bulk tissue *cis*-eQTL data with a concordant direction of effect, respectively (Supplementary Data 11). In line with the SAT cell-type-specific eQTLs, a greater number of cell-type level *cis*-eQTL variants were discovered in the SAT than VAT bulk tissue *cis*-eQTL data for all cell-types (adjusted *p* < 0.05) except for lymphoid cells, further supporting the tissue-specificity.

### Heritability of cardiometabolic and aging traits by *cis*-regions of eGenes

**Fig. 3:**
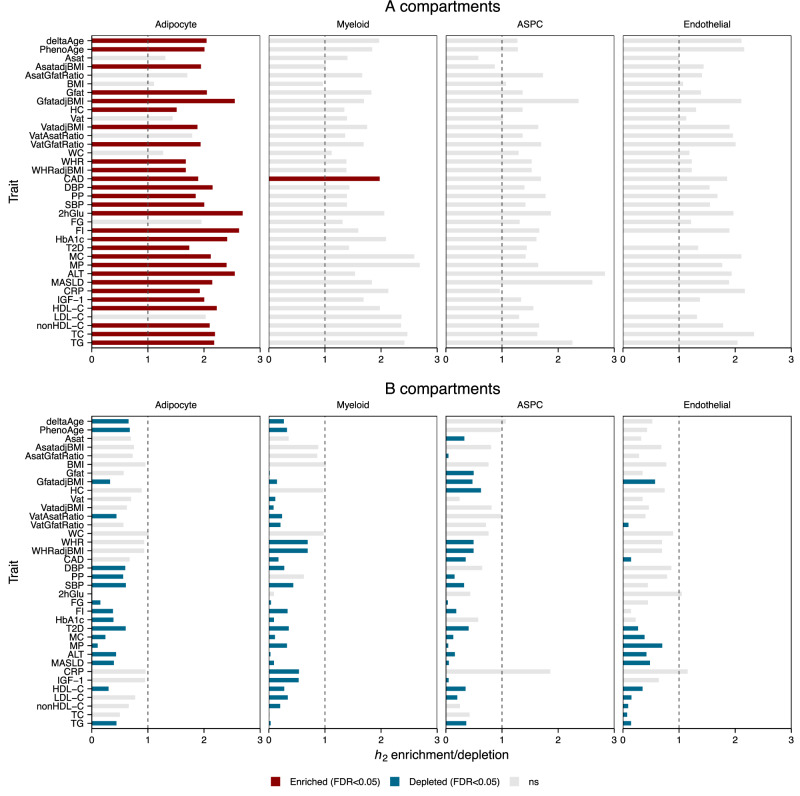
Heritability of cardiometabolic disease and biological aging traits by *cis*-regional variants of cell-type level eGenes is largely impacted by 3D genome organization. Bar plots showing significant (FDR < 0.05) enrichment and depletion of heritability for 36 CMD and biological aging traits by *cis*-regional variants of cell-type level eGenes residing in the A (top panel) or B (bottom panel) chromatin compartments. Heritability was measured by the heritability ratio (*h*_*2*_) using LDSC^40^. Vertical dashed lines at *h*_*2*_ = 1 indicate the threshold of no enrichment or depletion of heritability. Colors represent significant enrichment (FDR < 0.05 and *h*_*2*_ > 1), depletion (FDR < 0.05 and *h*_*2*_ < 1), and non-significant results. Only the results with *h*_*2*_ up to 3 are shown. The full heritability results and trait abbreviations are available in Supplementary Data 12 and 14. ASPC adipose stem and progenitor cells, FDR false discovery rate, ns not significant.

To assess relationship between eGenes and heritability of obesity-associated traits across the five SAT cell-types, we used linkage disequilibrium-score regression (LDSC)^40^ to quantify the proportion of heritability of 36 cardiometabolic disease (CMD) and biological aging traits explained by variants in the *cis*-regions of cell-type level eGenes. Without chromatin compartment stratification, no cell-type showed significant enrichment or depletion of heritability for any of the 36 traits (Supplementary Data 12, 13). As we had observed more enrichment in *cis-*regulatory elements and epigenomic features by lead *cis*-eQTL variants in A compartments (Fig. 1h), we also stratified *cis*-regional variants based on the chromatin compartments, in which their cell-type level eGenes reside, and repeated the heritability analysis. Out of 36 traits, we found significant enrichment of heritability (FDR < 0.05) for 28 traits by adipocyte eGenes in A compartments (Fig. 3 and Supplementary Data 12, 14). The heritability for coronary artery disease was also significantly enriched by myeloid cell eGenes in A compartments. In contrast, the *cis*-regional variants of eGenes in B compartments were broadly depleted for heritability. Specifically, we found that heritability for 33 traits showed significant depletion in at least one cell-type (FDR < 0.05). Using a relaxed FDR threshold (FDR < 0.10) added enrichment for five traits (all in A compartments) and depletion for ten traits (all in B compartments) (Supplementary Fig. 2 and Supplementary Data 14). Thus, *cis-*regions of adipocyte eGenes in A compartments account for a disproportionate share of CMD and biological aging traits than those in B compartments that are depleted for their heritability across all cell-types. Our results illustrate epigenomic chromatin compartmentalization as a potential mechanism for disease risk regulation and highlight adipocyte eGenes in A compartments as key contributors to CMD and biological aging risks in individuals with obesity.

### Colocalization with cardiometabolic disease- and aging-associated variants

**Fig. 4:**
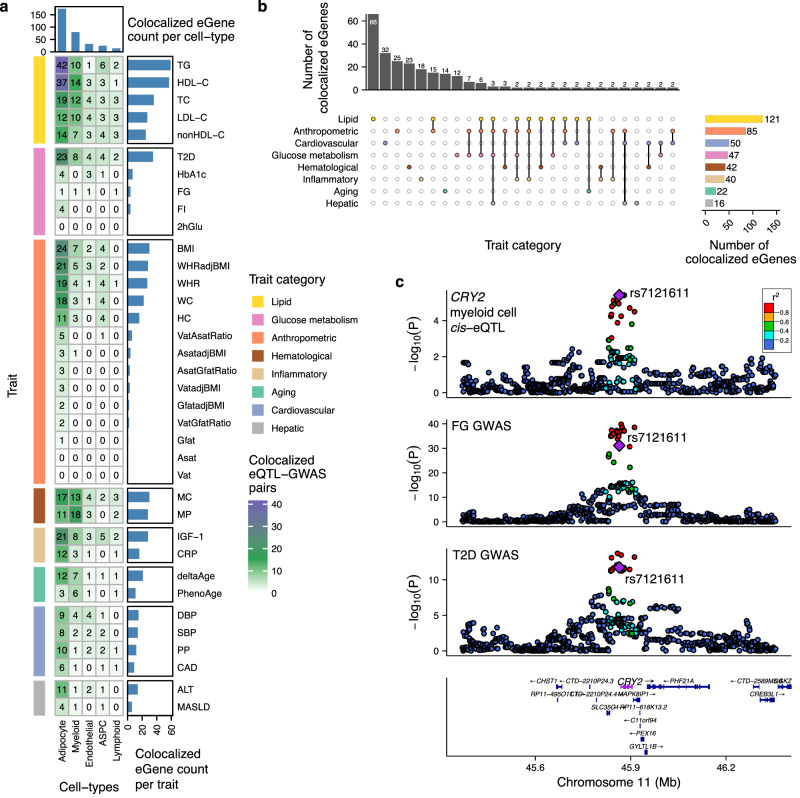
Cell-type level *cis*-eQTL mapping discovers regulatory variants underlying cardiometabolic disease and biological aging traits. **a** Heatmap showing the number of colocalized (PP4 ≥ 0.5) SAT cell-type level *cis*-eQTL and 36 cardiometabolic and biological aging trait genome-wide association study (GWAS) variant pairs. Vertical bar plots (top panel) represent the number of unique eGenes with colocalized *cis*-eQTL variants (i.e., colocalized eGenes) in at least one trait for each SAT cell-type. Horizontal bar plots (right panel) represent the number of colocalized eGenes in at least one cell-type for each trait. The full colocalization results and trait abbreviations are available in Supplementary Data 12 and 15. **b** Upset plot showing the number of shared and specific colocalized eGenes across the combinations of trait categories. The horizontal bar plots (right panel) indicate the total number of colocalized eGenes identified in each trait category. Only trait category combinations with at least two colocalized eGenes are shown. **c** LocusZoom plots of myeloid cell *cis*-eQTL variants for *CRY2*, fasting glucose (FG) GWAS, and type 2 diabetes (T2D) GWAS. The lead *cis*-eQTL variant, rs7121611, is indicated by a purple diamond. Colors represent linkage disequilibrium (LD), *r*^2^, with the lead *cis*-eQTL variant. The unadjusted *p*-values of the *cis*-eQTL variants were computed using two-sided tests as implemented in tensorQTL^27^. The unadjusted *p*-values of the GWAS variants were derived from the corresponding previously published GWAS studies, listed in Supplementary Data 12. ASPC adipose stem and progenitor cells, *CRY2* cryptochrome circadian regulator 2, eQTL expression quantitative trait loci, Mb megabase, *P* *p*-value, PP4 posterior probability of a shared single causal variant.

To connect cell-type level regulatory variants to disease risks, we performed Bayesian colocalization analysis between the cell-type level *cis*-eQTL and GWAS signals for 36 CMD and biological aging traits classified into eight broad trait categories (“Methods” and Supplementary Data 12, 15). We discovered 682 colocalized eQTL-GWAS pairs involving 279 unique genes in 33 out of 36 traits tested across the five SAT cell-types (Fig. 4a and Supplementary Data 15). In general, colocalizations and eGenes with colocalized *cis*-eQTL variants (referred to as “colocalized eGenes” hereinafter) were most frequent for traits that are directly relevant for the biological functions of SAT and obesity, including lipid traits triglycerides (TG; *n* = 59 colocalized eGenes) and high-density lipoprotein-cholesterol (HDL-C; *n* = 57), and anthropometric traits body mass index (BMI; *n* = 30) and waist-to-hip ratio adjusted for BMI (WHRadjBMI; *n* = 28). We also observed colocalizations for at least one trait in the aging, cardiovascular, glucose metabolism, hematological, hepatic, and inflammatory trait categories, supporting the broader role of SAT in CMDs and biological aging beyond fat storage and mobilization (Fig. 4a and Supplementary Data 15).

To gain insight into the regulatory mechanisms underlying the colocalized eGenes, we performed a transcription factor (TF) motif enrichment analysis within the *cis*-regions of the 279 colocalized eGenes using HOMER^41^. We observed two significantly (*p* = 1 × 10^−12^) enriched motifs, for AT-rich interactive domain-containing protein 5a (Arid5a) and highly divergent homeobox (Hdx). Previous mouse studies have shown that Arid5a regulates adipogenesis through a counter-regulation with Ppar-γ2 and that Arid5a deficiency in mice leads to adult-onset obesity^42^. In addition, Arid5a is known to promote inflammation through stabilizing proinflammatory mRNAs^43–45^. Overall, the enrichment of the Arid5a binding motif in the *cis*-regions of colocalized eGenes suggests transcriptional regulation by Arid5a and downstream effectors of Arid5a-mediated adipogenesis and inflammatory regulations as one of the potential biological mechanisms underlying cardiometabolic and biological aging risks under obesity.

Of the 279 colocalized eGenes, 192 (69%) were identified only in one trait category, likely indicating distinct molecular mechanisms underlying the diseases in SAT (Fig. 4b and Supplementary Data 15). Among the eight trait categories, lipids had the most trait category-specific colocalized eGenes accounting for 66 out of 192 (34%) genes (Fig. 4b). For example, an adipocyte *cis*-eQTL variant of *CORO1C* colocalized with GWAS signals for all five lipid traits but not with traits from other categories (Supplementary Fig. 3 and Supplementary Data 15). Similarly, a myeloid cell *cis*-eQTL variant of the cryptochrome circadian regulator 2, *CRY2*, colocalized only with glucose metabolism traits, including fasting glucose (FG) and type 2 diabetes (T2D) (Fig. 4c and Supplementary Data 15). Cryptochromes are circadian clock genes that have been shown to regulate macrophage metabolism and inflammatory immune responses, which can lead to conditions such as T2D^46–48^. In contrast, an adipocyte *cis*-eQTL variant of *MLXIPL*, which encodes a key transcription factor for regulation of both glucose and lipid metabolism^49,50^, colocalized with at least one trait in five categories (Supplementary Fig. 4 and Supplementary Data 15).

**Fig. 5:**
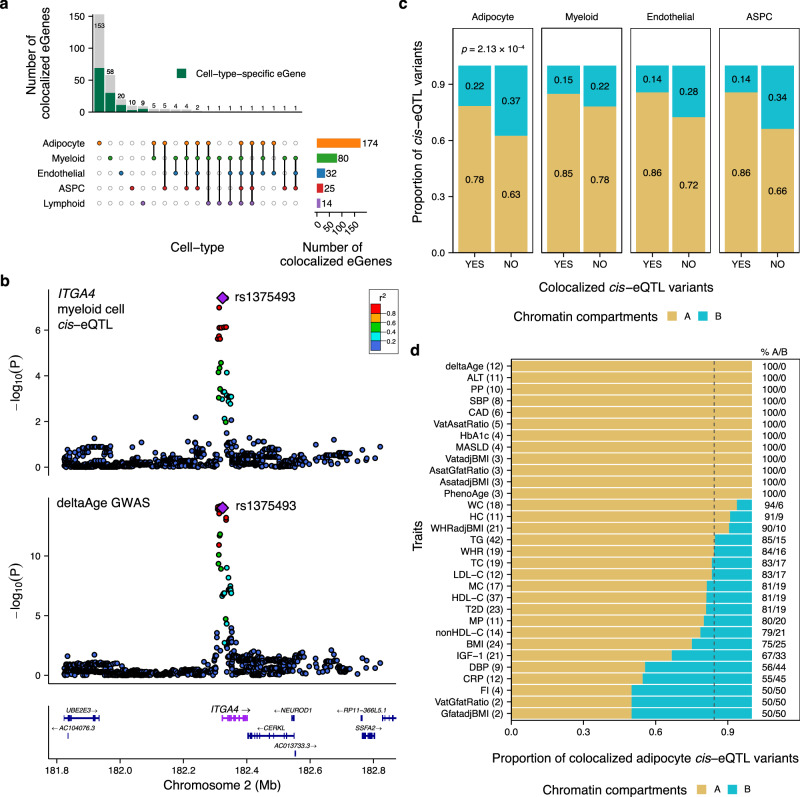
Adipocyte *cis*-eQTL variants colocalized with cardiometabolic disease and biological aging traits are enriched in the chromatin A compartments. **a** Upset plot showing the number of cell-type-shared and cell-type-specific eGenes with a colocalized *cis*-eQTL variant (i.e., colocalized eGenes) across the combinations of SAT cell-types. The horizontal bar plots (right panel) display the total number of colocalized eGenes identified in each cell-type. Dark green color on the bars in the top panel represents the number of cell-type-specific eGenes identified using mashr^38^. **b** LocusZoom plots of the myeloid cell *cis*-eQTL variants for *ITGA4* and accelerated biological aging (deltaAge) GWAS. The lead *cis*-eQTL variant, rs1375493, is indicated by a purple diamond. Colors represent linkage disequilibrium (LD), *r*^2^, with the lead *cis*-eQTL variant. The unadjusted *p*-values of the *cis*-eQTL variants were computed using two-sided tests as implemented in tensorQTL^27^. The unadjusted *p*-values of the GWAS variants were derived from the corresponding previously published GWAS studies, listed in Supplementary Data 12. **c** Proportion of the colocalized cell-type level *cis*-eQTL variants that reside in the A or B chromatin compartments. Enrichment of the colocalized *cis*-eQTL variants in A compartments was evaluated using a hypergeometric test after applying Bonferroni correction across the four cell-types. **d** Stacked bar plots showing proportion of colocalized adipocyte *cis*-eQTL variants that are in A versus B chromatin compartments for each trait. The numbers on the right indicate percent of colocalized *cis*-eQTL variants in A/B compartments. Only the traits with more than one colocalized eQTL-GWAS pair are shown. The vertical dashed line represents the average proportion (0.84) of lead *cis*-eQTL variants landing in the adipocyte A compartments. Trait abbreviations are available in Supplementary Data 12. ASPC adipose stem and progenitor cells, eQTL expression quantitative trait loci, *ITGA4* Integrin alpha-4, Mb megabase, *P* *p*-value.

We next assessed cell-type specificity of the eQTL-GWAS colocalizations. Overall, we observed that 90% of the colocalized eGenes are cell-type-specific (Fig. 5a). In more detail, we found the greatest number of colocalized eGenes in adipocytes, totaling 174 unique genes across all traits, of which 153 are only discovered in adipocytes (i.e., cell-type-specific colocalization) (Fig. 5a and Supplementary Data 15). Similarly, we discovered 58, 20, 10, and 9 colocalized eGenes only in myeloid, endothelial, ASPCs, and lymphoid cells, respectively, demonstrating that the CMD and biological aging risk contributions from SAT are largely cell-type-specific (Fig. 5a). For example, we discovered adipocyte-specific colocalization of *APOL6* eQTL variant with TG and HDL-C signals (Supplementary Fig. 5 and Supplementary Data 15). *Apol6*, a member of the apolipoprotein L gene family, has been shown to inhibit lipolysis in mouse adipocytes through a direct interaction with *Plin1*, a key protein that coats lipid droplets in adipocytes^51^. In contrast, colocalization of Integrin alpha-4 encoding *ITGA4* and all aging and hematological trait signals are specific to myeloid cells (Fig. 5b and Supplementary Data 15), emphasizing the role of SAT inflammation in these traits. We also observed that only a modest percent (30–56%) of the eGenes with cell-type-specific colocalization are cell-type-specific eGenes from the mashr analysis (Fig. 5a), suggesting that not all cell-type-specific colocalizations are observed with cell-type-specific eGenes. In line with this, many eGenes with cell-type-specific colocalization are eGenes in multiple cell-types (Fig. 5a), suggesting that many genes with *cis*-eQTL variants in several cell-types only show colocalization in only one cell-type. This seems to indicate that distinct regulatory variants may act on the same gene in different SAT cell-types, potentially due to context-specific epigenomic regulation, and contribute to different disease outcomes.

We further compared the cell-type level colocalizations with the bulk SAT eQTL-GWAS colocalizations from AdipoExpress^13^ for the 25 shared traits between the studies (“Methods”). Of the 499 colocalized cell-type level eQTL-GWAS pairs across these 25 traits, the majority (334; 67%) were not detected in the bulk analysis (Supplementary Data 16). Specifically, the replication rate of colocalized eQTL-GWAS pairs was highest for endothelial cells (45%), followed by adipocytes (37%), lymphoid cells (28%), myeloid cells (25%), and ASPCs (16%). The 334 colocalizations we discovered regulate 168 unique eGenes. Notably, 89% (150/168) of these eGenes are cell-type-specific colocalized eGenes in our analysis with 36 traits (Supplementary Data 16), suggesting that the bulk signals were either masked by cellular averaging or absent in the relatively healthy bulk SAT cohorts^13^. For the replicated pairs, we provide the cell-type of origin for the eQTL signals (Supplementary Data 16), adding valuable biological information to bulk tissue colocalizations^13^. Altogether, these results highlight the power of cell-type level *cis*-eQTL analysis to uncover previously unknown regulatory variants and genes underlying CMDs while revealing cell-types of origin for previously known colocalizations.

Finally, motivated by stronger heritability of obesity-associated traits in *cis*-regions of adipocyte eGenes residing in the A compartments (i.e., epigenomic active compartments) than in B compartments (Fig. 3), we further investigated whether the colocalized lead *cis*-eQTL variants preferentially reside in active chromatin that comprises on average 45% of the genome across the SAT cell-types. Among the *cis*-eQTL variants with chromatin compartment annotations available, we observed a higher proportion of the colocalized variants in A compartments (mean 84%; range 78–86% across the SAT cell-types) compared with the non-colocalized *cis*-eQTL variants (mean 70%; 63–78%) (Fig. 5c). In particular, the colocalized adipocyte *cis*-eQTL variants were significantly enriched in adipocyte A compartments (78% in A versus 22% in B; hypergeometric test; adjusted *p* = 2.13 × 10^−4^), while the other four cell-types showed nominal trends (nominal* p*-value 0.05–0.13) (Fig. 5c). Among the 31 CMD and biological aging traits with more than one colocalized eQTL-GWAS pair in adipocytes, 16 traits have a higher percent of lead *cis*-eQTL variants landing in the adipocyte A compartments than the average (84%), including 12 CMD and biological aging traits with 100% of colocalized variants residing in the adipocyte A compartments (Fig. 5d). Together with the heritability analysis results, these results underscore the relevance of distinct 3D genome organization as a regulatory mechanism of disease risk variants and highlight adipocyte eGenes in A compartments as key contributors to CMD and accelerated biological aging risk in individuals with obesity.

### Partitioned T2D PRS built of colocalized variants predicts both T2D and BMI

To further assess broader clinical applicability of our colocalized results, we constructed a partitioned polygenic risk score (PRS) for T2D in UKB using 32 non-redundant lead T2D GWAS variants that colocalize with cell-type level *cis*-eQTLs. We observed that the partitioned PRS significantly predicts T2D diagnosis (*p* = 4.73 × 10^−26^), with Nagelkerke’s pseudo-R^2^ of 0.056 and an AUC of 0.680 (CI = 0.676–0.684). Including the partitioned PRS improved the predicted power from the covariates-only model, yielding an incremental Nagelkerke’s pseudo-R^2^ of 9.74 × 10^−4^ and increasing the AUC by 1.53 × 10^−3^. These results are comparable to the incremental Nagelkerke’s pseudo-R^2^ (4.54 × 10^−3^) and increase in AUC (6.15 × 10^−3^) achieved with a “full” GWAS T2D PRS built from 197 independent T2D GWAS variants, indicating that the colocalized variants capture a substantial fraction of T2D genetic susceptibility.

As the partitioned PRS comprises T2D GWAS variants that are colocalized with *cis*-eQTL variants in individuals with obesity, we sought to assess the relationship of this PRS with BMI. To this end, we tested for the association between the partitioned T2D PRS and BMI. We observed a significant positive association (beta = 0.02, *p* = 5.35 × 10^−45^), and thus the partitioned T2D PRS predicts both T2D and BMI. This result demonstrates complex polygenic correlations between T2D and BMI, where the underlying pathways of the colocalized T2D genes likely have pleiotropic effects on both traits.

To further corroborate the genetic effect on T2D at the colocalized loci, we also assessed the main effect of the partitioned T2D PRS on T2D after accounting for BMI by including it as a covariate. We discovered that the partitioned T2D PRS is still a significant predictor of T2D (*p* = 6.10 × 10^−44^), with Nagelkerke’s pseudo-R^2^ of 0.157 and an AUC of 0.787 (CI = 0.783–0.790). Including the partitioned PRS improved the predicted power from the covariates-only model that includes BMI, yielding an incremental Nagelkerke’s pseudo-R^2^ of 1.83 × 10^−3^ and increasing the AUC by 1.16 × 10^−3^, suggesting that the polygenic effects on T2D are not fully explained by BMI. These results are in line with the fact that the colocalized loci were discovered using eQTLs from individuals with obesity and that obesity is a main risk factor for T2D. Taken together, we highlight how cell-type level genetic and epigenomic regulatory variants prioritize functionally informed variant subsets that predict disease relevant to obesity.

### T2D colocalized eGenes interact with known drugs

To further investigate the T2D colocalized eGenes and their therapeutic potential, we cross-referenced the 35 unique eGenes that colocalized with T2D GWAS signals across the five cell-types against the Drug-Gene Interaction Database^52^ (DGIdb) v5.0.11. We observed that seven out of the 35 unique eGenes (20%) are known to interact with drugs (Supplementary Data 17). Among these seven genes there is an adipocyte eGene, *MARK3*. A previous study has shown that the siRNA-mediated knockdown of *Mark3* in mouse-derived 3T3-L1 adipocytes increases insulin-stimulated GLUT4 translocation, resulting in an insulin hypersensitivity^53^. Thus, the agents targeting MARK3 may have a therapeutic potential for T2D upon further investigation. Some of the notable drugs from the analysis include cilostazol and pentoxifylline. These are inhibitors of phosphodiesterase, such as phosphodiesterase 3A, which is encoded by an adipose stem and progenitor cell (ASPC) eGene, *PDE3A*, and known to hydrolyze cAMP^54^. In mouse-derived 3T3-L1 preadipocytes, cAMP contributes to an increased transcription of *Pparg*, a master regulator of adipogenesis, through activation of protein kinase A (PKA)^55^. Thus, inhibition of PDE3A may contribute to increase in preadipocyte differentiation. In line with this, cilostazol has been shown to promote differentiation of 3T3-L1 preadipocytes to adipocytes via activation of PPARG and increase insulin sensitivity^56^. Similarly, reduction in insulin resistance was observed with pentoxifylline therapy in a previous randomized study^57^. Together, these results suggest that cell-type level colocalized T2D eGenes provide potential biologically anchored therapeutic targets of T2D for individuals with obesity.

## Discussion

We discovered SAT cell-type level regulatory variants and their epigenomic landscape that contribute to genetic predisposition of CMDs and accelerated biological aging in individuals with obesity. We characterized *cis*-eQTLs in the main SAT cell-types with epigenomic data and colocalized them with cardiometabolic and accelerated biological aging traits, highlighting adipocyte eGenes in A compartments as key contributors to CMD and biological aging risk in individuals with obesity. We further found that the *cis*-eQTLs that colocalize with CMD and biological aging GWAS variants are largely cell-type-specific and not identified in the previous SAT bulk colocalization study^13^. Next, we demonstrate that a partitioned PRS for T2D, constructed using the cell-type level colocalized T2D GWAS variants, significantly predicts the T2D status in the UK Biobank with a substantial association with BMI, thus marking the T2D risk in SAT cell-types. These results demonstrate that cell-type level genetic and epigenomic regulatory variants can elucidate key subsets of variants that predict T2D.

Utilizing SAT single-cell multi-omics datasets, this study advances the field beyond prior SAT bulk *cis*-eQTL and colocalization studies^11–13^ in several ways. First, we discovered previously unknown cell-type level *cis*-eQTLs and linked them to CMD and accelerated biological aging risks through colocalization analyses. Concurrently, we resolved the underlying cell-type of action for many of the known bulk SAT *cis*-eQTLs and previously known colocalizations. Second, we investigated individuals with obesity unlike the previous studies that comprised predominantly healthy individuals. Thus, we capture obesity-relevant regulatory variants and their links to CMD and accelerated aging. Third, we performed colocalization analysis with eight additional traits that were not previously tested in the SAT bulk colocalization analysis^13^, including two aging (accelerated aging and biological aging), two hematological (monocyte counts and percentage), two inflammatory (c-reactive protein and insulin-like growth factor-1), and two hepatic (alanine transaminase and metabolic dysfunction-associated steatotic liver disease) traits. This is important because these eight traits are known to associate with obesity and related CMDs^2,10,58,59^, and we discovered 40 previously unreported colocalized eQTL-GWAS pairs across the five SAT cell-types. Thus, we provide more comprehensive insight into the regulatory mechanisms underlying CMDs and accelerated aging. Fourth, we integrated human SAT cell-type level DNA methylation and three-dimensional (3D) genome organization data to improve localization of CMD variants by uncovering their epigenomic regulatory architecture underlying disease regulatory mechanisms. We show that cell-type level *cis*-eQTL variants are preferentially localized in the transcriptionally active (A) chromatin compartments and enriched for *cis*-regulatory elements and epigenomic features. Furthermore, we found that *cis*-regional variants of eGenes in adipocyte A compartments are significantly enriched for CMD and accelerated biological aging trait heritability. In line with this finding, we showed that these active compartments are also enriched for *cis*-eQTL variants colocalized with the trait-associated risk variants, especially in adipocytes. These results provide further insights into colocalized CMD and accelerated aging variants by characterizing their cell-type level 3D chromatin architecture.

Increasing numbers of cell-type level eQTL studies in other tissues have shown that many *cis*-eQTLs are cell-type-specific and context-dependent^7,14–18^. In line with these studies, we discovered that in individuals with obesity, most *cis*-eQTL variants in SAT are also cell-type-specific. Particularly, more *cis*-eQTL variants were identified in adipocytes than in other SAT cell-types even after accounting for the difference in their abundance. A large fraction of these adipocyte *cis*-eQTLs is also adipocyte-specific. As the primary SAT cell-type with a multitude of biological functions, ranging from fat storage to adipokine secretion, adipocytes show early signs of obesity-associated pathologies^60^. Thus, the greater number of identified *cis*-eQTLs in adipocytes may reflect increased regulation of adipocytes in response to obesity.

Interestingly, adipocyte lead *cis*-eQTL variants residing in inactive (B) chromatin compartments were enriched for promoters unlike the other cell-types. This enrichment result suggests that while the overall chromatin region is inactive in B compartments, genetic regulations of local promoters stay present. These promoters may reflect a poised state with regulatory potential that could be activated under a specific dynamic condition, such as in response to obesity. Future studies with additional chromatin profiling under various conditions would allow for a deeper understanding of the regulatory promoters in B compartments.

Adipose tissue, particularly obese adipose tissue, is increasingly being recognized as a potential driver of biological aging^10,58,61^, suggesting that its genetic regulation may also contribute to the risk of accelerated biological aging. In our data, we identified 33 colocalized cell-type level eQTL-GWAS signals for two biological aging traits, including myeloid *cis*-eQTL variants for α4 integrin, encoding *ITGA4*. A previous mouse study has shown that α4 integrin on macrophages interacts with vascular cell adhesion molecule 1 (VCAM-1) on adipocytes to form macrophage-adipocyte adhesion, marked by increased *Itga4*-sufficient macrophages and *Vcam1* expression in SAT of mice with diet-induced obesity^62^. The sustained macrophage-adipocyte interaction consequently reduced *Ucp1* expression in adipocytes, which in turn inhibits beige adipogenesis in obesity^62^. Decrease in beige adipocytes is one of the key features of aging^10,63^, suggesting that the genetic regulation of *ITGA4* in macrophages may contribute to individuals’ biological aging.

Colocalized eQTL-GWAS signals could be utilized to identify individuals with increased genetic predisposition to diseases and discover therapeutic targets. We found that the partitioned T2D PRS constructed using these colocalized GWAS variants significantly predict T2D status. The eGenes of T2D GWAS colocalized variants include SH2B adaptor protein 1, encoded by the *SH2B1* gene. Previous knockout of *Sh2b1* in mice peripheral tissues, including the adipose tissue, resulted in an impaired insulin signaling followed by severe hyperglycemia, hyperinsulinemia, and glucose intolerance^64,65^. SH2B1 directly interacts with insulin receptors, IRS-1 and IRS-2, to promote insulin receptor catalytic activity through phosphorylation of tyrosine residues, thereby enhancing insulin sensitivity^64,65^. Notably, we also observed that *SH2B1* interacts with several second-generation antipsychotics including amisulpride, aripiprazole lauroxil, clozapine, olanzapine, paliperidone, quetiapine fumarate, and risperidone. Second-generation antipsychotics are reported to have adverse effects of increased bodyweight and increased risk of diabetes, hyperglycemia, and dyslipidemia as well as new-onset diabetes in the absence of obesity^66,67^. These converging findings suggest that the interaction between *SH2B1* and second-generation antipsychotic in adipose tissue may contribute to the observed T2D risk. Our results along with this known biology from animal models and drug interactions further indicate the role of *SH2B1* in T2D pathogenesis.

Even with the identified cell-type level *cis*-eQTLs and colocalizations, our study has some limitations. First, the lack of individuals with a healthy range of BMI limits our ability to define, which of the identified *cis*-eQTL variants are context-specific. However, given that previous SAT bulk tissue studies^11–13^ are largely limited to healthy individuals, it is important to also characterize genetic regulations within the context of obesity. Our findings are still relevant for the large proportion of the population with obesity (BMI ≥ 30), comprising ~40% of US adults, with similar rates reported elsewhere^68^. Second, the sample size of our SAT snRNA-seq and cell-type level SAT epigenomic datasets constrained our ability to study rare cell-types and perform sex-stratified analysis. Although we are missing regulatory effects of rare cell-types, the five cell-types selected for the main analysis represent the major cellular lineages present in SAT. Similarly, the used cell-type level SAT epigenomic data were derived from five females. Although the sample size is common for such a high-complexity single-cell assay, it captures only a small part of inter-individual variability. However, as a substantial proportion of the snRNA-seq data used in this study are from females (64%), the cell-type level snm3C-seq data remain informative for the broad epigenomic localization of the cell-type level *cis*-eQTLs. The limited sample size of snRNA-seq data also prevented sex-stratified analysis. Furthermore, sex-stratified GWAS summary statistics for the partitioned heritability and colocalization analyses were not available for all investigated traits. Thus, we focused on the datasets that included both sexes and adjusted for sex to maximize power and comprehensively test larger numbers of CMD traits. Overall, further expanding future sample sizes will improve generalizability of our findings to rare cell-types and additional populations and enable discovery of sex-specific *cis*-regulatory variants and epigenomic features. Lastly, we performed colocalization using coloc^69^ that assumes a single causal variant per locus. To allow for multiple causal variants within a locus, we performed stepwise conditional analysis to identify conditionally independent signals at each locus in the same way as in the previous large SAT bulk tissue colocalization study^13^. This multi-signal detection design has also been used previously^11–13,70,71^.

In summary, we conducted SAT cell-type level *cis*-eQTL mapping and colocalization analysis and then integrated these with single cell epigenomic data to identify cell-type level regulation for CMDs and accelerated biological aging. We discovered 291 colocalized regulatory variants for 33 CMD and accelerated biological aging traits, of which 208 have not been identified in prior bulk tissue colocalization studies. We also characterized their underlying cell-type level 3D chromatin architecture, overall localizing disease mechanisms and providing a highly valuable resource for the discovery of therapeutic targets for obesity-related comorbidities and accelerated biological aging.

## Methods

### Ethics

The Kuopio Obesity Surgery Study (KOBS) was approved by the Ethics Committee of the Northern Savo Hospital District (54/2005, 104/2008, and 27/2010). The longitudinal Roux-en-Y versus one-anastomosis gastric bypass (RYSA) study was approved by the Helsinki University Hospital Ethics Committee (HUS/1706/2016). The Tilkka study was approved by the Helsinki University Hospital Ethics Committee (HUS/1039/2019). The UKB study was approved by the North West Multi-centre Research Ethics Committee (21/NW/0157). All participants in these studies provided a written informed consent, and no compensation was provided to the participants. All research was conducted in accordance with the principles of the Declaration of Helsinki.

### Study cohorts and omics data types

We used single nucleus RNA-sequencing (snRNA-seq) data of 127 subcutaneous adipose tissue (SAT) biopsies from Finnish individuals with obesity who participated in the KOBS^72,73^ (*n* = 59; 32 females; mean age = 49.1 years (SD 9.7)) and RYSA^21,74^ (*n* = 68; 49 females; mean age = 46.3 years (SD 7.9)) bariatric surgery cohorts.

Genotype data of all 127 individuals were available for *cis*-eQTL mapping. We included variants on autosomes present in both cohorts and excluded variants with minor allele frequency (MAF) < 10% in the combined 127 individuals, which resulted in 4,589,727 variants for the cell-type level *cis*-eQTL mapping. To prevent confounding from relatedness, we confirmed that no individuals were related up to 3rd degree using KING^75^ v2.3.2. We also analyzed imputed genotype data from European-origin unrelated individuals of the UK Biobank cohort (UKB)^76,77^, and single nucleus methyl-3C sequencing (snm3C-seq) data of 5 SAT biopsies from Finnish individuals^20^. Detailed description of the study cohorts, genotype data quality control, imputation, and SAT snRNA-seq data generation and processing can be found in Supplementary Methods.

### Integration of the human SAT snRNA-seq datasets

We combined the KOBS and RYSA SAT snRNA-seq datasets using Seurat^78^ v4.3.0.1 to merge the two Seurat objects and to perform variable gene selection, scaling, and PCA on the merged object, as described in Supplementary Methods. For data visualization, we integrated the datasets on batch using Harmony^79^ v1.0.3, which accounted for the differences in gene expression by both batch and cohort. The final Uniform Manifold Approximation and Projection (UMAP) visualization of the integrated datasets was created using the ‘RunUMAP’ function in Seurat with reductions from Harmony. Detailed description of data processing and quality control can be found in Supplementary Methods.

For a high coverage *cis*-eQTL mapping and to match the cell-type annotations with the epigenomic SAT snm3C-seq data^20^, we combined cell-types of shared lineage (i.e., natural killer cells and T cells as lymphoid cells and macrophages and monocytes as myeloid cells) and limited the *cis*-eQTL mapping to the five most prevalent cell-types, adipocytes, adipose stem and progenitor cells (ASPCs), endothelial cells, lymphoid cells, and myeloid cells. For every cell-type, we created pseudobulk counts of gene expression per sample by correcting the raw counts for library size and log-transforming the counts using scran^80^ and then aggregating across cells by taking the mean log-transformed counts of each gene^81^. We included genes on autosomes with mean count >0.1 in at least 50% of the samples, which resulted in 7625 genes for adipocytes, 5331 for ASPCs, 5443 for endothelial, 3845 for lymphoid, and 5828 for myeloid cells. We then performed rank-based inverse normal transformation on the pseudobulk counts.

### Cell-type level *cis*-eQTL mapping using tensorQTL

For every cell-type, we conducted *cis*-eQTL mapping by fitting a linear regression model using tensorQTL^27^ v1.0.9 (*cis*_nominal) with variants within ±500 kb from ends of the genes. To account for technical variations between samples, we performed PCA on the pseudobulk expression. We optimized the number of expression principal components (PCs) included as covariates by selecting the number of PCs that maximized *cis*-eGenes discovered, resulting in 19, 21, 23, 23, and 21 PCs for adipocytes, ASPCs, endothelial, lymphoid, and myeloid cells, respectively. We confirmed that sex is significantly correlated with at least one expression PC for all five cell-types (Supplementary Fig. 6). Next, we employed the permutation mode in tensorQTL (*cis*) with 10,000 permutations to calculate gene level q-values from the beta distribution-extrapolated empirical *p*-values of the top associated variant-gene pairs. To identify significant eGenes with at least one significant *cis*-eQTL variant, we applied a false discovery rate (FDR) threshold of 0.05. All significant *cis*-eQTL variants associated with an eGene were then identified using the nominal *p*-value threshold calculated from the permutation-based beta distribution using tensorQTL.

### Identification of conditionally independent *cis*-eQTL signals

To identify additional independent genetic signals affecting the same gene, we used the stepwise regression procedure employed in tensorQTL^27^ v1.0.9 (*cis*_independent) with the same covariates as in the primary *cis*-eQTL mapping. This approach identifies multiple causal variants per gene by conditioning on previously discovered variants in iterative forward and backward selection steps. Briefly, a forward and backward stage *cis*-eQTL mapping was performed correcting for covariates and all previously discovered variants. For each iteration, a lead variant with a beta distribution-extrapolated empirical *p*-value at the significance threshold was considered as an independent signal. Next, we generated summary statistics with conditioned* p*-values for eGenes with more than one conditionally independent signal for the downstream colocalization analyses. We extracted each lead *cis*-eQTL variant and performed *cis*-eQTL mapping, as described above with the same covariates, while conditioning on all other lead *cis*-eQTL variants for that gene. For eGenes with just one lead variant, we used the marginal *p*-values from the original *cis*-eQTL mapping.

### Comparison of *cis*-eQTLs across SAT cell-types

To test whether adipocytes showed significantly more eGenes than expected based on cell abundance, we calculated the ratio of eGenes to the mean number of nuclei of the samples for each cell-type. We then computed a modified z-score comparing the adipocyte ratio to other cell-types and assessed significance using a one-tailed test.

To further verify the observed greater number of eGenes in adipocytes, we performed a down-sampling analysis by randomly subsampling adipocyte nuclei to match the counts of endothelial and lymphoid cells for each donor. If an individual had less adipocytes than endothelial or lymphoid cells, all available adipocytes were included. *Cis*-eQTL mapping was then repeated using tensorQTL^27^ v1.0.9 as described above with the pseudobulk expression of the down-sampled adipocytes.

Next, we searched for differences in the median magnitude of *cis*-eQTL effect size across the five SAT cell-types using two-sided Kruskal-Wallis test. The *cis*-eQTL effect sizes were measured using tensorQTL^27^ v1.0.9.

### Comparison of SAT cell-type level *cis*-eQTLs with bulk SAT *cis*-eQTLs

We compared the cell-type level primary lead *cis*-eQTL variants with bulk SAT *cis*-eQTL variants from AdipoExpress^13^. We evaluated proportion of cell-type level lead *cis*-eQTL variants that were also *cis*-eQTLs in AdipoExpress, focusing on gene-variant pairs that were tested in both datasets. We considered a variant as a *cis*-eQTL in AdipoExpress based on a *p*-value threshold of 1 × 10^−6^.

### Cell-type level *cis*-eQTL mapping using SAIGE-QTL

To assess robustness of our cell-type level *cis*-eQTLs from tensorQTL^27^ across different methodological frameworks, we repeated *cis*-eQTL mapping by fitting a Poisson mixed model implemented in SAIGE-QTL^26^ v0.3.4 with single-cell-level gene counts. For comparability, we tested the same genes and variants as in the original analysis and used the same pseudobulk expression PCs as detailed above. We performed single variant association tests for all genes, for which a null Poisson mixed model was successfully fitted and obtained gene-level *p*-values using the ACAT test implemented in SAIGE-QTL. To identify significant eGenes, we applied an FDR threshold of 0.05 on the gene-level *p*-values.

### Characterization of cell-type level *cis*-eQTL variants and eGenes

We characterized eGenes of primary lead *cis*-eQTL variants based on probability of being loss-of-function (LoF) intolerant (pLI)^28^. The LoF metrics were downloaded from gnomAD^82^ v4.1.0 and pLI of the canonical transcript was used for each gene. We classified all genes that were tested for *cis*-eQTL mapping as LoF intolerant (pLI ≥ 0.9), tolerant (pLI ≤ 0.1), or ambiguous (0.1 < pLI < 0.9)^28^. Then, we used the Chi-square test to assess differences in proportions of the LoF intolerant genes between the genes with and without significant *cis*-eQTL variants (i.e., eGenes vs non-eGenes) in each cell-type. To define significance, we applied a Bonferroni correction based on the number cell-types (total 5 tests) and used an adjusted *p*-value < 0.05. Next, LoF intolerant eGenes were tested for enrichments of the Gene Ontology^83^ biological processes, Reactome pathways^84^, and WikiPathways^85^ using WebGestaltR^86^ v0.4.6 with all discovered eGenes as background for each cell-type. Enrichments with the Benjamini-Hochberg corrected *p* < 0.05 were considered significant. Lastly, we searched for differences in the median magnitude of lead *cis*-eQTL variant effect sizes for LoF intolerant and tolerant eGenes using the Wilcoxon rank sum test. We applied a Bonferroni correction based on the number cell-types (total 5 tests) and used an adjusted *p*-value < 0.05 to define significance.

Next, we tested for enrichment of lead *cis*-eQTL variants in epigenetic regulatory elements, including imputed promoter and enhancer chromatin states of adipocyte sample E023 and adipose nuclei sample E063 in the 25-state ChromHMM model from the Roadmap Epigenomics project^32,33^, open chromatin peaks of preadipocytes and 14-day differentiated adipocytes from ATAC-seq data^34^, and corresponding cell-type level differentially methylated regions (DMRs; hyper- and hypo-) from SAT snm3C-seq^20^. The adipocyte lead *cis*-eQTL variants were tested for the enrichments in promoters and enhancers of both adipocyte sample E023 and adipose nuclei sample E063. For the lead *cis*-eQTL variants of the four other cell-types, we used promoters and enhancers of only adipose nuclei sample E063 given that this sample represents the entire adipose tissue. The enrichment in preadipocyte and adipocyte ATAC peaks were only tested for ASPC and adipocyte lead *cis*-eQTL variants, respectively, due to the lack of data for the other cell-types. The lead variants of genes without a significant *cis*-eQTL were used as the background. Employing GARFIELD^87^ v2 with default parameters, we first performed greedy pruning (linkage disequilibrium (LD) *r*^2^ > 0.1) on the lead variants of all genes tested in the *cis*-eQTL analysis using the UKB genotype data of randomly selected 40,000 unrelated Europeans as the LD reference panel. Next, the LD pruned variants and their LD proxies (*r*^2^ > 0.8) were annotated based on their overlap with the epigenomic regulatory elements. Finally, we quantified enrichments using odds ratio (OR) by applying logistic regression while accounting for MAF, distance to nearest transcription start site, and number of LD proxies. We applied a Bonferroni correction based on the number cell-types and epigenetic annotations tested (total 24 tests) and used an adjusted *p*-value < 0.05 to define cell-type level *cis*-eQTLs to be significantly enriched (OR > 1) or depleted (OR < 1).

To compare enrichments in epigenetic regulatory elements by cell-type level chromatin compartments, we repeated the enrichment analysis of epigenetic annotations described above separately for the cell-type level lead *cis*-eQTL variants residing in the active (A) or inactive (B) compartments of the corresponding cell-types. We used the lead *cis*-eQTL variants in each chromatin compartment as the test set and the lead variants of genes without a significant *cis*-eQTL as the background. The enrichment analysis was limited to the cell-types with corresponding SAT cell-type level chromatin compartment data (adipocytes, ASPCs, endothelial cells, and myeloid cells). We applied a Bonferroni correction based on the number cell-types, epigenetic annotations, and compartments tested (total 40 tests) and used an adjusted *p*-value < 0.05 to define cell-type level *cis*-eQTLs to be significantly enriched (OR > 1) or depleted (OR < 1).

Lastly, cell-type level eGenes in the A compartments of the corresponding cell-type were tested for enrichments of the Gene Ontology^83^ biological processes, Reactome pathways^84^, and WikiPathways^85^ using WebGestaltR^86^ v0.4.6 as described above. We considered enrichments with the Benjamini-Hochberg corrected *p*-value < 0.05 as significant.

### Cross cell-type sharing of *cis*-eQTLs

We assessed cell-type sharing of *cis*-eQTL effects using the multivariate adaptive shrinkage (mash)^38^ method (v0.2.50). We combined z-scores of all tested variants for eGenes with at least one *cis*-eQTL variant in at least one cell-type. For variant-gene pairs not tested in specific cell-types, we set z-scores to 0 and standard error to 1 × 10^6^. Next, we selected top *cis*-eQTL variants with a max z-score across all cell-types for each gene to estimate data-driven covariance matrices. We estimated default canonical covariance matrices in a subset of 250,000 randomly selected variant-gene pairs that were tested across all cell-types. Finally, we fitted the mash model and computed posterior summaries and local false sign rate (LFSR).

We considered an eGene to be cell-type-shared if it had at least one significant *cis*-eQTL variant with LFSR < 0.05 in more than one cell-type, and to be cell-type-specific if it had a *cis*-eQTL variant (LFSR < 0.05) only in one cell-type. Sharing of eGenes between two cell-types were evaluated for all pairwise combinations of five cell-types. For variant level sharing, we first identified a top *cis*-eQTL variant (most significant LFSR < 0.05) for a gene in each cell-type. If the gene had more than one top variant across cell-types, we performed LD pruning (*r*^2^ > 0.1) using PLINK^88^ v1.9 (--indep-pairwise 200 100 0.1) with UKB as the LD reference panel. Next, we assessed sharing of the top *cis*-eQTL variants where a variant was considered shared if it had the same direction of effect, and the estimated effect size was within a factor of 2 when compared to the cell-type with the greatest effect size. The top *cis*-eQTL variants that were shared with at least one cell-type were considered as cell-type-shared and others as cell-type-specific. We compared the median MAF and magnitude of effect sizes between the cell-type-shared and -specific top *cis*-eQTL variants using the Wilcoxon rank sum test.

### Evaluating tissue-specificity of SAT cell-type-specific and cell-type level *cis*-eQTLs

To evaluate tissue-specificity of the SAT cell-type-specific *cis*-eQTLs, we compared the cell-type-specific top *cis*-eQTL variants with SAT and visceral adipose tissue (VAT) bulk *cis*-eQTL variants from the European subset of GTEx analysis V8^39^. Focusing on gene-variant pairs that were tested in our study and both in the GTEx SAT and VAT analyses, we evaluated the proportion of the lead *cis*-eQTL variants that were also *cis*-eQTLs in GTEx based on a *p*-value threshold of 1 × 10^−6^. We then repeated the comparison for the cell-type level lead *cis*-eQTL variants of the five SAT cell-types. Next, for each variant set, we assessed whether the proportion of variants replicated in the bulk SAT data is greater than those in bulk VAT data using the one-sided McNemar’s test implemented by the ‘mcnemarExactDP’ function of the R package exact2x2 v1.6.5. We applied a Bonferroni correction based on the number of variant sets tested (total 6 tests) and used an adjusted *p*-value < 0.05 to define significance.

### Identification of conditionally independent GWAS signals

To enable accurate colocalization analyses between *cis*-eQTL and GWAS variants, we first identified conditionally independent GWAS signals for each trait and then computed conditioned summary statistics for loci with multiple independent signals. A detailed list of the GWAS summary statistics of the 36 cardiometabolic disease (CMD) and biological aging traits used in this study is included in Supplementary Data 12. We used summary statistics of GWASs conducted in European ancestry only or primarily European ancestry for all downstream analyses. For the two biological aging traits, we conducted GWASs using genotype and phenotype data from 331,481 (177,802 females; mean age = 56.9 years (SD 8.0)) unrelated British individuals in UKB^76,77^. Biological age was estimated using the PhenoAge formula^89^, and aging acceleration was defined as the difference between biological and chronological age^89^. We applied rank-based inverse normal transformations to both traits and used BOLT-LMM^90^ v2.3.6 to perform the GWASs on the normalized values. Chronological age, chronological age^2^, sex, genotyping array, testing center, and top 20 genetic PCs were included as covariates, and only variants passing MAF > 1% and an imputation score *r*^2^ > 0.3 were tested.

For colocalization analyses, we identified conditionally independent GWAS signals and computed conditioned summary statistics for loci with more than one independent signal using GCTA^91^ v1.94.3. For traits with conditionally independent lead variants reported by the original authors (BMI, WHR, WHRadjBMI, CAD, SBP, DBP, PP, FG, FI, 2hGlu, HbA1c, and ALT), we identified loci containing multiple reported lead variants within 500 kb of each other. For these multi-signal loci, we estimated conditional summary statistics for each lead variant using GCTA cojo-cond with default parameters and UKB genotype data from 40,000 randomly selected unrelated Europeans as the LD reference panel. For loci with one lead variant, we used the marginal summary statistics for the colocalization analyses. For traits where conditionally independent lead variants were not reported by the original authors, we identified them using a systematic approach, as described previously^13^. Briefly, we defined each locus as a lead variant with marginal *p*-value < 5 × 10^−8^ and its 500 kb flanking regions. We then merged loci, the lead variants of which were within 1 Mb of each other, continuing this process iteratively until no further merging was possible. Within each merged locus, we identified conditionally independent lead variants using GCTA cojo-slct with MAF ≥ 1%, collinearity < 0.5, and otherwise default parameters. The UKB genotype data were used as the LD reference panel, as described above. For loci with multiple conditionally independent lead variants, we estimated conditional summary statistics for each lead variant using GCTA cojo-cond with the same parameters. For loci with a single lead variant, we used marginal summary statistics.

### Partitioned heritability analyses of cell-type level eGenes

To assess whether cell-type-specific eGenes contribute disproportionately to trait heritability, we applied stratified linkage-disequilibrium (LD) score regression (LDSC)^40,92^ v1.0.1 to partition the heritabilities of the 36 CMD and biological aging GWAS traits in UKB^76,77^. We calculated separate LD scores for variants within the full *cis*-regions (± 500 kb around each gene body) of the cell-type level eGenes and their chromatin compartment-stratified subsets. For computational efficiency, we used DNA-level genotype data from a randomly selected 20% subset of the unrelated British individuals from UKB (*n* = 76,758) and restricted analyses to variants with MAF > 5%^40^. First, we calculated LD scores for all variants within the *cis*-regions of genes expressed in each cell-type and for variants landing in the *cis*-regions of cell-type level eGenes. We then estimated the overall heritability, as well as the conditional per-SNP heritability contributions relative to the background of the expressed genes in the cell-type using the LD scores and corresponding GWAS summary statistics. To assess significant heritability contributions, we applied two-sided *z*-tests on the per-SNP heritability estimates^40^ with FDR correction across 5 cell-types and 36 traits, using FDR < 0.05 to define significance.

For cell-types with corresponding cell-type level chromatin compartment data (adipocytes, ASPCs, endothelial cells, and myeloid cells), we performed additional analyses stratified by active (A) or inactive (B) compartments. We calculated LD scores for variants within the *cis*-regions of eGenes residing in each compartment and estimated heritability contributions and conditional per-SNP heritability contributions relative to the background of the expressed genes in the cell-type, as described above. We applied FDR correction across four cell-types, two chromatin compartments, and 36 traits and used FDR < 0.05 to define significant heritability contributions.

### Colocalizing *cis*-eQTL and trait-associated GWAS variants

To identify shared causal variants underlying both gene expression changes and disease risk, we performed Bayesian colocalization between conditionally independent *cis*-eQTL and GWAS variants of 36 CMD and biological aging -associated traits using coloc^69^ v5.1.0 (coloc.abf) with default parameters. We restricted analyses to the loci where lead *cis*-eQTL and GWAS variants were located within 500 kb and were in moderate-to-high LD (*r*^2^ ≥ 0.5), ensuring biological plausibility of shared causation. LD calculations were performed using PLINK^88^ v1.9 with the UKB LD reference panel, as described above. For loci with single conditionally independent lead variants, we applied colocalization using marginal summary statistics of *cis*-eQTL and GWAS. If multiple conditionally independent lead *cis*-eQTL and/or GWAS variants were present at a locus, we used their respective conditional summary statistics computed as described above. We identified significantly colocalized loci using the estimated posterior probability of a shared single causal variant (PP4) ≥ 0.5, consistent with the SAT bulk *cis*-eQTL meta-analysis and colocalization study^13^, to which we directly compared our colocalization results. We used the same PP4 threshold to ensure methodological comparability between the two studies.

We compared our cell-type level colocalization results with previous findings from bulk SAT study^13^ for 25 CMD-associated traits (Supplementary Data 16). For each trait, we considered our colocalized cell-type level eQTL-GWAS pairs to be replications of bulk tissue findings if the same lead *cis*-eQTL variants or their LD proxies (*r*^2^ ≥ 0.5) colocalized with the same GWAS variants or their LD proxies (*r*^2^ ≥ 0.5). Colocalized eGenes and/or GWAS variants that differed from the bulk tissue study were classified as previously unreported cell-type-specific colocalized pairs.

### TF motif enrichment analysis for colocalized eGenes

We identified transcription factor (TF) motifs enriched in the *cis*-regions of the 279 colocalized eGenes using the ‘findMotifsGenome.pl’ program in Hypergeometric Optimization of Motif EnRichment (HOMER)^41^. We defined a *cis*-region as 2 kb upstream and 1 kb downstream of the transcription start site (TSS) for each gene.

### Partitioned polygenic risk analysis using colocalized cell‑type level *cis*‑eQTL and T2D GWAS variants

To assess the clinical utility of our cell-type level colocalization findings, we constructed partitioned polygenic risk scores in UKB^76,77^ for T2D using T2D lead GWAS variants that colocalized with cell-type level lead *cis*-eQTL variants. To ensure reliable PRS construction and avoid sample overlap bias that could inflate results^93^, we used previously published T2D GWAS summary statistics from the DIAGRAM consortium^94^, which excluded UKB participants, for the effect size weights of variants. We first performed LD-clumping of the colocalized GWAS variants based on their GWAS *p*-values using PLINK^88^ v1.9, applying an *r*^2^ threshold of < 0.1 and window size of 250 kb to ensure independence. The resulting set of independent variants (*n* = 32 variants) was used to compute the partitioned PRSs for T2D with the PLINK ‘--score’ function, where each PRS represented the average of risk allele dosages weighted by the GWAS effect sizes.

We evaluated predictive performance by using the partitioned T2D PRS to predict T2D status^73,95^ with logistic regression. We included the top 20 genetic PCs, testing center, genotyping array, chronological age, chronological age^2^, and sex as covariates in the model to control for population structure and demographic factors. The significance of the partitioned T2D PRS effect was tested using a Wald test with a *p*-value < 0.05 as the significance threshold. We quantified predictive gain by calculating the change in AUC contributed by the partitioned T2D PRS relative to a covariate‑only baseline model. To examine specificity of our results, we repeated the PRS analysis using all independent T2D GWAS variants (*n* = 197), as described above.

To assess whether the partitioned T2D PRS is associated with BMI, we predicted BMI using the T2D PRS with logistic regression. We included the same covariates as described above except BMI as an outcome. The significance of the partitioned T2D PRS effect on BMI was tested using a Wald test with a *p*-value < 0.05 as the significance threshold.

Finally, we reassessed the main effect of the partitioned T2D PRS on T2D by including BMI as a covariate in addition to the other covariates described above. We quantified the predictive gain by calculating the change in AUC contributed by the partitioned T2D PRS relative to a covariate‑only baseline model that included BMI and tested for the significance of the partitioned T2D PRS effect on T2D using a Wald test with a *p*-value < 0.05 as the significance threshold.

### Cross-referencing colocalized T2D eGenes against a drug target database

To assess whether the colocalized T2D eGenes interact with known drugs, we cross referenced the 35 unique colocalized eGenes that were identified across the five cell-types against the Drug-Gene Interaction Database^52^ (DGIdb) v5.0.11. The list of gene-drug interactions and their interaction scores were obtained from https://dgidb.org.

### Reporting summary

Further information on research design is available in the Nature Portfolio Reporting Summary linked to this article.

## Supplementary information

Supplementary Information

Description of Additional Supplementary Files

Supplementary Data 1-17

Reporting Summary

Transparent Peer Review file

## Data Availability

The full cell-type level *cis*-eQTL summary level data, generated using tensorQTL and SAIGE-QTL, and the GWAS summary statistics of the biological aging traits are available in Zenodo^96^ [https://zenodo.org/records/18880304]. Previous publications and their links to the summary statistics of the cardiometabolic disease trait GWASs are listed in Supplementary Data 12. The KOBS SAT snRNA-seq data are available in the NIH Gene Expression Omnibus (GEO) under accession number GSE302701. The RYSA SAT snRNA-seq data are available in GEO under accession number GSE274778^21^. The SAT snm3C-seq data are available in GEO under accession number GSE297267^20^. The adipocyte ATAC-seq data are available in GEO, under accession number GSE269929^34^. Data from the UK Biobank were used in this study under UK Biobank Application Number 33934. UK Biobank data are available for bona fide researchers through the application process [https://www.ukbiobank.ac.uk/learn-more-about-uk-biobank/contact-us].

## Code availability

The code used in this study is available at GitHub [https://github.com/seunghyuktlee/SAT_snRNA_eQTL_2026]^97^.
