# Supplementary material for: Adipose single cell epigenome and transcriptome localize genetic risk for cardiometabolic disease and accelerated aging
Source: Nat Commun. 2026 Apr 20;17:5469. doi: 10.1038/s41467-026-72248-4 (PMC13284207; doi:10.1038/s41467-026-72248-4)
Supplement: Supplementary file 1 — Supplementary Information [file 41467_2026_72248_MOESM1_ESM.pdf]

Supplementary Information

Supplementary Methods

Study cohorts

KOBS SAT snRNA-seq cohort

Finnish individuals with obesity undergoing bariatric surgery were recruited in the University of Eastern Finland and Kuopio University Hospital, Kuopio, Finland to participate in the Kuopio Obesity Surgery Study (KOBS), as described previously[1,2]. In this study, we used genotype and phenotype data and matching single nucleus RNA-sequencing (snRNA-seq) data of subcutaneous adipose tissue (SAT) biopsies from 59 KOBS participants. All participants provided a written informed consent, and the KOBS study was approved by the Ethics Committee of the Northern Savo Hospital District, in accordance with the Declaration of Helsinki.

RYSA SAT snRNA-seq cohort

In this study, we used genotype and phenotype data and matching snRNA-seq data of SAT biopsies from 68 Finnish individuals with obesity who participated in the longitudinal Roux-en-Y versus one-anastomosis gastric bypass (RYSA) study, as described previously[3–5]. The study participants were recruited in the Helsinki University Hospital, Helsinki, Finland and they underwent either Roux-en-Y gastric bypass or One-anastomosis gastric bypass bariatric surgery[3]. We used snRNA-seq data of SAT biopsies collected at the time of operation[4]. All participants provided a written informed consent, and the RYSA study was approved by the Helsinki University Hospital Ethics Committee, in accordance with the Declaration of Helsinki.

Tilkka SAT snm3c-seq cohort

We used single nucleus methyl-3C sequencing (snm3C-seq) data of SAT biopsies collected from five Finnish females (mean age = 44.4 years (SD 4.78)) who underwent abdominal liposuction at Tilkka Hospital[6]. All participants provided a written informed consent, and the study was approved by the Helsinki University Hospital Ethics Committee, in accordance with the Declaration of Helsinki.

UK Biobank

We used genotype and phenotype data from unrelated individuals of European-origin of the UK Biobank cohort (UKB), collected across 22 assessment centers[7,8]. The Applied Biosystems UK BiLEVE Axiom Array or Applied Biosystems UK Biobank Axiom Array were used to generate genotype data. The imputation of the genotype data was performed against the Haplotype Reference Consortium and the merged UK10K and 1000 Genomes phase 3 reference panels. Data from UKB were accessed under application 33934.

Genotype quality control and imputation

We used existing genotype data from the KOBS[2] and RYSA[4] cohorts generated using the Infinium Global Screening Array-24 v1 (Illumina). The genotype data from the two cohorts were separately quality controlled using PLINK[9] v1.9 where individuals with missingness > 2%, unmapped, strand ambiguous, and monomorphic SNPs, and variants with missingness > 2% and Hardy-Weinberg Equilibrium (HWE) p-value < 10^{-6} were excluded[2,4]. Additionally, the reported sex of all individuals was cross-checked against the inferred biological sex imputed using the '--sex-check' function in PLINK v1.9. We used these existing high-quality genotype data to

perform genotype imputation against the TOPMed reference panel version r3[10] using Minimac4[11] and haplotype phasing using Eagle[12] v2.4 on the TOPMed imputation server. In each cohort, we removed imputed genotype variants with imputation score $r^2 < 0.3$ and HWE p-value $< 10^{-6}$.

Nuclei isolation and generation of human SAT snRNA-seq dataset

We used the existing human SAT snRNA-seq data of 59 and 68 frozen SAT biopsies from the KOBS and RYSA[4,5] participants, respectively. Briefly, within each cohort, we first designed batches with a pool of 7 to 8 frozen SAT biopsies per batch using random assignments that minimized associations with phenotypic traits, including age, sex, BMI, and type 2 diabetes (T2D) diagnosis status. For every batch, we pooled approximately 100 mg of each biopsy and used the gentleMACS Dissociator (Miltenyi Biotec) and the nuclei isolation protocol for 10x snRNA-seq[4]. We measured the concentration and evaluated the overall quality of the isolated nuclei using the Countess II FL Automated Cell Counter after staining nuclei from each batch with the Hoechst stain. Nuclei were then loaded into the 10x Chromium Chip for library construction using the Single Cell 3' v3.1 chemistry. All libraries were evaluated using an Agilent Bioanalyzer and sequenced together for each cohort on the NovaSeq X Plus 25B (KOBS) or NovaSeq 6000 S4 (RYSA)[4,5].

Processing of the human SAT snRNA-seq datasets

The human SAT snRNA-seq data from the KOBS and RYSA cohorts were processed separately following our data processing and quality control pipeline[4,5]. Briefly, for each batch, we aligned the raw FASTQ format file against the GRCh38 human genome reference and GENCODE v42 annotations[13] using STAR[14] v2.7.10b with the '--soloFeatures GeneFull' option to account for

full pre-mRNA transcripts. To remove empty droplets and droplets with high amounts of ambient RNA, we used DIEM[15] v2.4.0 with a UMI cutoff of 500 to define debris, k-means clustering of 50 for the initialization step, and otherwise default parameters. The UMI cutoff of 500 was originally empirically selected based on the UMI distribution plots across all nuclei that would ensure high-quality nuclei while reducing empty and ambient RNA droplets[4,5]. Next, we used Seurat v4.3.0.1 to remove low-quality droplets with the number of unique molecular identifiers (UMIs) ≤ 500, number of unique genes detected (nFeatures) ≤ 200, percentage of reads mapped to the mitochondrial genome (%mito) ≥ 10, and spliced RNA ≥ 75%. The gene counts of the remaining droplets were log-normalized using the default scaling factor of 10,000. Then we performed a principal component analysis (PCA) on the gene counts scaled to mean 0 and variance 1 for the top 2,000 variable genes, calculated excluding mitochondrial and ribosomal genes. To remove contaminated counts from the remaining droplets, we employed DecontX[16] from celda R package v1.14.2 with the previously removed low-quality droplets as the background and the Seurat cluster assignment, obtained using the standard Louvain algorithm with the first 30 PCs and a clustering resolution of 0.5. The remaining gene counts were then used to recalculate UMIs, nFeatures, and %mito. Additional low-quality nuclei with UMIs ≤ 500, UMIs ≥ 30,000, nFeatures ≤ 200, and %mito ≥ 10 were excluded. We identified the individual of origin for the nuclei using demuxlet v2 from popscle software tool[17] with '--min-MQ 30' and otherwise default parameters and employing imputed genotype data. Lastly, we removed nuclei that were labeled as doublets or ambiguous as well as filtered out additional doublets, identified using DoubletFinder v2.0.3[18] with the most optimal pK value that maximized the mean-variance normalized bimodality coefficient from the pN-pK parameter sweeps on a subset of 10,000

nuclei. Only genes with ≥ 3 raw counts in ≥ 3 nuclei across all batches were kept for the downstream steps.

We merged all remaining high-quality nuclei in the batches within each cohort using Seurat[19] v4.3.0.1 and performed gene count normalization, variable gene selection, scaling, and PCA, as described above in the merged data. Next, we accounted for batch driven differences in gene expression using Harmony[20] v1.0.3 and employed the reductions from Harmony to perform clustering with a resolution of 0.5. The clusters were annotated as one of five broad cell-types: adipocytes, adipose stem and progenitor cells (ASPCs), lymphoid cells, myeloid cells, or vascular cells, using SingleR[21] v1.8.1 with the SAT single cell and snRNA-seq data from the previously published adipose tissue atlas[22] as a reference. For every broad cell-type, we performed variable gene selection, scaling, PCA, Harmony integration on batch, and clustering (Harmony dimensions 20–30 and resolutions 0.2–0.3) to annotate the main cell-types, as described above. The cell-type annotations from the broad cell-types were next carried back to the full dataset, and their marker genes were identified using a Wilcoxon rank sum test from the 'FindAllMarkers' function in Seurat (Bonferroni adjusted p-value < 0.05) with logFC.threshold = 0.25 and min.pct = 0.25.

Generation and processing of the human SAT snm3C-seq dataset

We used the cell-type level methylation and chromatin compartment mapping from the human SAT snm3C-seq data that is detailed in the previous publication[6]. Briefly, in situ chromatin conformation capture was performed using Arima Genomics Arima-HiC Kit and fluorescence-activated nuclei sorting, and library preparation were performed using the snmC-seq3 workflow

(https://www.protocols.io/view/snm3c-seq3-kqdg3x6ezg25). We sequenced the snmC-seq3 libraries using an Illumina NovaSeq 6000 with a read length of 150 bp and mapped the sequenced reads against the GRCh38 human genome reference using Taurus-MH[23]. Next, we performed quality control on the data based on estimated non-conversion rate of mCCC% < 0.015, global mCG% > 0.5, global mCH% < 0.15, total number of interaction contacts >100,000 and <500,000, and at least one intra-chromosome contact present in each autosome after removing reads with either end mapped to the ENCODE blacklist regions[6].

To cluster the snm3C-seq data, we first separately derived low-dimensional embeddings for each modality. For the methylation modality, we first calculated hypomethylation score per cell and for each 5 kb bin after removing ENCODE blacklist regions and binarized the score with nominally significant entries as 1 and rest as 0. Next, latent semantic indexing was performed on the inverse-log-document-frequency transformed matrix, as implemented in the ALLCools package[24] v1.0.23, to retrieve the mCG profile embedding. We removed the first dimension due to its high correlation with sequencing depth. Concurrently, for the chromosome conformation modality, we first imputed the contact matrix of each cell at 100 kb resolution using scHiCluster[25] v1.3.5 with pad = 1. Then, we projected all intra-chromosome contacts between 100 kb and 10 Mb on autosomes to a low-dimensional space using singular value decomposition. Lastly, we used Harmony[20] on the concatenated joint embedding of the top 10 dimensions from the two modalities to remove sample level batch effect followed by constructing k-NN graphs (k = 25) and performing Leiden consensus clustering. Each cluster was annotated to a cell-type using negative correlations between mCG and expression of the known SAT marker genes from matching snRNA-seq data of the Tilkka cohort[6].

Constructing cell-type level methylation profiles

The cell-type level mCG profiles from the SAT snm3C-seq data were constructed by first aggregating number of CG methylated counts and total coverage across cells of each cell-type. Next, we identified differentially methylated sites (DMSs) by finding genomic regions with distinct mCG patterns across cell-types using methylPy[26,27], as implemented in the ALLCools package. Each DMS was assigned as hypo-methylated, neutral, or hyper-methylated based on whether the normalized deviation away from the mean methylation level (i.e., fitted residual) fell below the 0.4, between the 0.4 and 0.6, or above the 0.6 quantile of its chromosome-wide background, respectively. We then merged DMSs within 250 bp with correlated methylation fractions (Pearson correlations > 0.8) to obtain differentially methylated regions (DMRs) and assigned differential methylation states (i.e., hypo-methylated, neutral, or hyper-methylated) to each DMR by averaging the fitted residuals of the merged DMSs. Lastly, we removed DMRs containing only one DMS or without any hyper- or hypo-methylation state assignment and those overlapping ENCODE blacklist regions.

Constructing cell-type level chromatin compartments

The cell-type level chromatin compartments from the SAT snm3C-seq data were constructed by first merging the scHiCluster imputed 100 kb contact matrices per chromosome across cells of each cell-type to obtain pseudobulk conformation profiles. Only 100 kb bins with a coverage < 99th percentile and above twice the 50th percentile minus the 99th percentile were kept, resulting in a removal of poorly mapped regions. We then normalized the cell-type level pseudobulk conformation profiles by the distance between the contacts and converted to correlation matrices

using dcHiC[28] v2.1. Lastly, we performed PCA on the cell-type level correlation matrices per chromosome and heuristically selected PC that maximized the absolute correlation with TSS and CpG density as the compartment scores using dcHiC. If needed, we flipped the sign of the scores to ensure that regions with positive scores correspond to more active (A) compartments. Lastly, we performed quantile normalization on the compartment scores.

Supplementary Figures

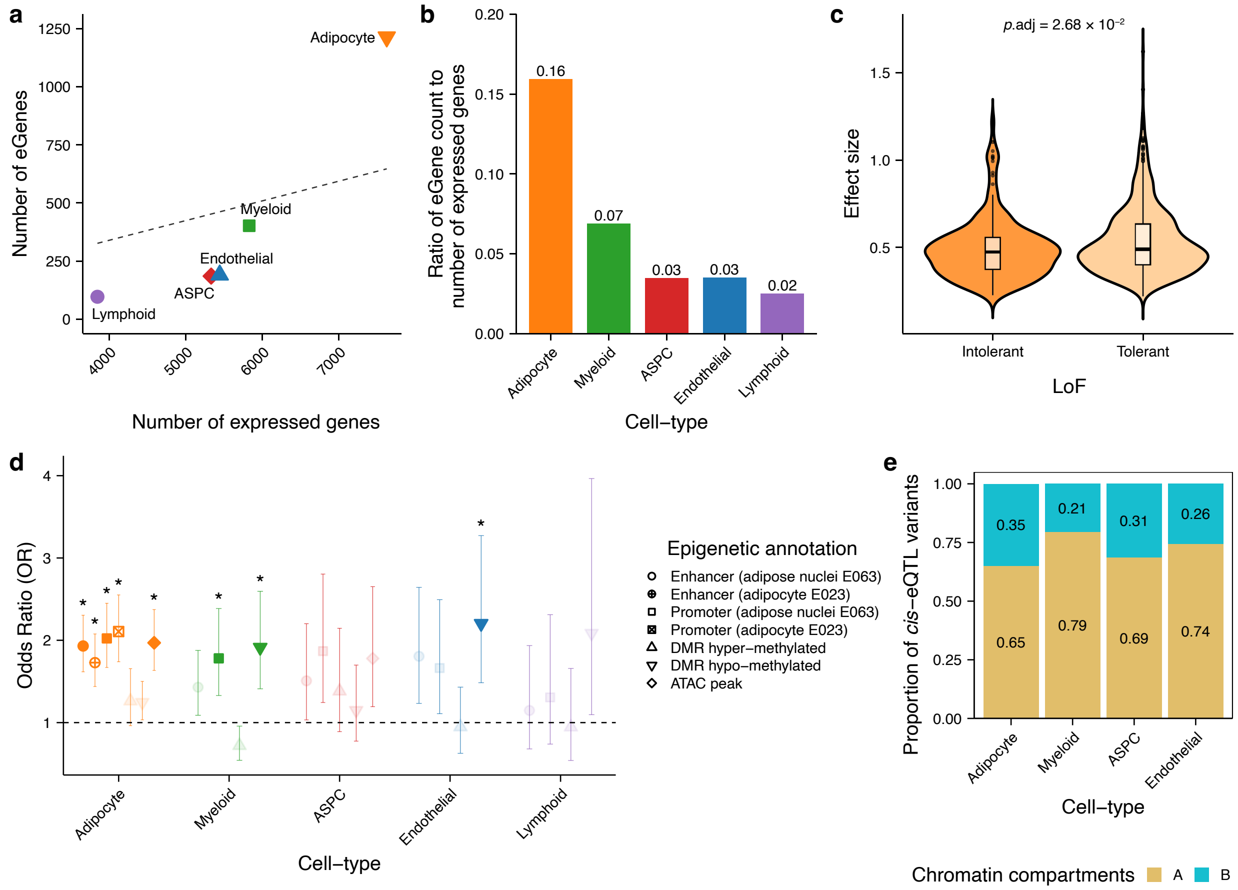

Supplementary Figure 1. Characterization of SAT cell-type level *cis*-eQTLs. a Scatter plot showing the number of cell-type level expressed genes tested for *cis*-eQTL mapping and eGene count of the corresponding cell-type. The dashed line represents the linear regression trend line of the number of expressed genes and eGenes. **b** Bar plots showing the ratio of eGenes to the number of cell-type level expressed genes. **c** Violin plots with inner boxplots showing the effect sizes of adipocyte lead *cis*-eQTL variants for loss-of-function (LoF) intolerant (n = 186) vs tolerant (n = 629) genes. Center lines of the boxes indicate the median magnitude of the variant effect size of the adipocyte lead *cis*-eQTL variants. Boxes range from the 25th–75th percentiles, whiskers show 1.5× the interquartile range (IQR), and black dots represent outliers. The median magnitude of the variant effect size of the adipocyte lead *cis*-eQTL variants was compared using

two-sided Wilcoxon rank sum test. **d** Enrichment of primary lead *cis*-eQTL variants in adipose promoters and enhancers, adipocyte and preadipocyte assay for transposase-accessible chromatin sequencing (ATAC) peaks, and SAT cell-type level differentially methylated regions (DMRs; hyper- and hypo-) (see Methods). Dots represent the odds ratios (OR), error bars 95% confidence intervals of the OR, dashed lines OR = 1, colors cell-type, shapes epigenetic annotation, and asterisks and opacity significant enrichments using two-sided test from GARFIELD[29] after Bonferroni correction. Exact p-values and the four different *n* values for each of the 24 enrichment tests are available in Supplementary Data 8. **e** Proportion of the lead *cis*-eQTL variants residing in A vs B chromatin compartments for each cell-type. Colors represent chromatin compartments. ASPC indicates adipose stem and progenitor cells; eQTL, expression quantitative trait loci; and *p*.adj, Bonferroni adjust p-value.

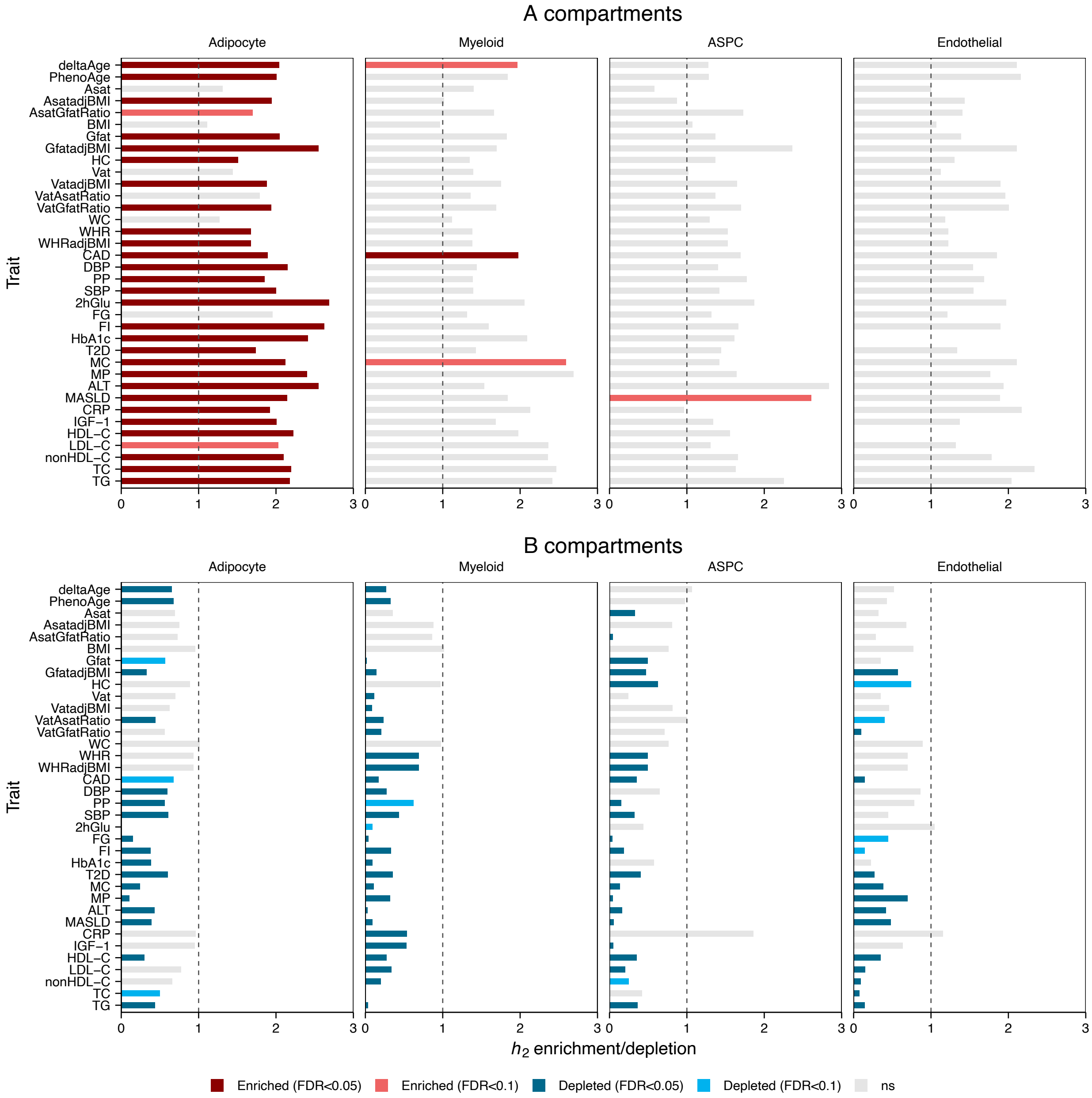

Supplementary Figure 2. Relaxing significance threshold reveals heritability of additional cardiometabolic disease and biological aging traits. Bar plots showing enrichment and depletion of heritability for 36 cardiometabolic disease (CMD) and biological aging traits by the *cis* regional variants of the cell-type level eGenes residing in the A (top panel) or B (bottom panel) chromatin compartments. Heritability was measured by the heritability ratio (h_2) using linkage disequilibrium-score regression (LDSC)[30]. Vertical dashed lines at $h_2 = 1$ indicate the

threshold of no enrichment or depletion of heritability. Colors represent significant enrichment ($h_2 > 1$ and FDR < 0.05 or 0.1), depletion ($h_2 < 1$ and FDR < 0.05 or 0.1), and non-significant results. Only the results with h_2 up to 3 are shown. The full heritability results and trait abbreviations are available in Supplementary Data 12 and 14.

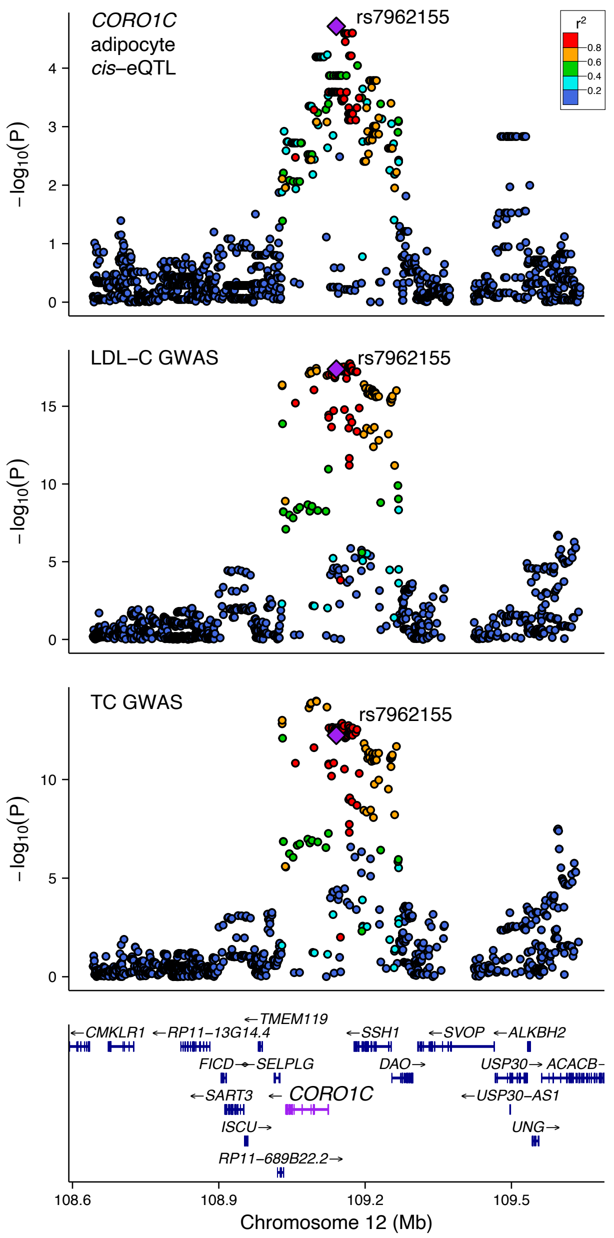

Supplementary Figure 3. Adipocyte *cis*-eQTL variant, rs7962155, regulating *CORO1C* is colocalized with all five lipid traits. LocusZoom plots of adipocyte cell *cis*-eQTL variants for *CORO1C*, high-density lipoprotein-cholesterol (HDL-C) GWAS, low-density lipoprotein-cholesterol (LDL-C) GWAS, non-high-density lipoprotein-cholesterol (nonHDL-C) GWAS, total cholesterol (TC) GWAS, and triglyceride (TG) GWAS. The lead *cis*-eQTL variant, rs7962155, is indicated by a purple diamond. Colors represent linkage disequilibrium (LD), r^2, with the lead

cis-eQTL variant. The unadjusted p-values of the *cis*-eQTL variants were computed using two-sided tests as implemented in tensorQTL[31]. The unadjusted p-values of the GWAS variants were derived from the corresponding previously published GWAS studies, listed in Supplementary Data 12. *CORO1C* indicates Coronin 1C; GWAS, genome-wide association study; eQTL, expression quantitative trait loci; Mb, megabase; and P, p-value.

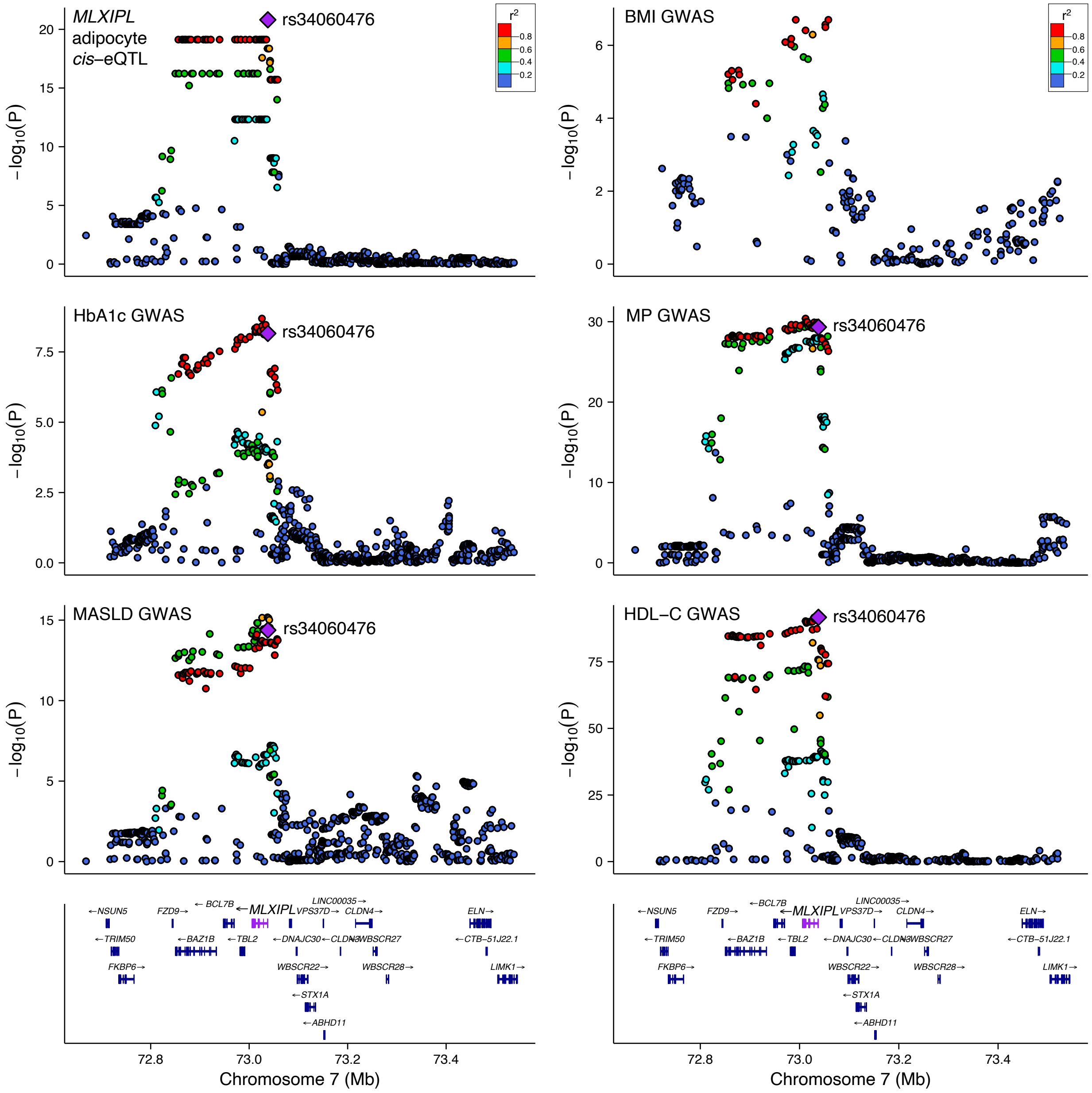

Supplementary Figure 4. Adipocyte *cis*-eQTL variant, rs34060476, of *MLXIPL* is colocalized with traits from five different cardiometabolic trait categories. LocusZoom plots of adipocyte cell *cis*-eQTL variants for *MLXIPL*, body mass index (BMI) GWAS, HbA1c (glycated hemoglobin) GWAS, monocyte percentage (MP) GWAS, metabolic dysfunction-associated steatotic (MASLD) GWAS, and high-density lipoprotein-cholesterol (HDL-C) GWAS. The lead *cis*-eQTL variant, rs34060476, is indicated by a purple diamond. Colors

represent linkage disequilibrium (LD), r^2, with the lead *cis*-eQTL variant. The unadjusted p-values of the *cis*-eQTL variants were computed using two-sided tests as implemented in tensorQTL[31]. The unadjusted p-values of the GWAS variants were derived from the corresponding previously published GWAS studies, listed in Supplementary Data 12. GWAS indicates genome-wide association study; eQTL, expression quantitative trait loci; Mb, megabase; *MLXIPL*, MLX Interacting Protein Like; and P, p-value.

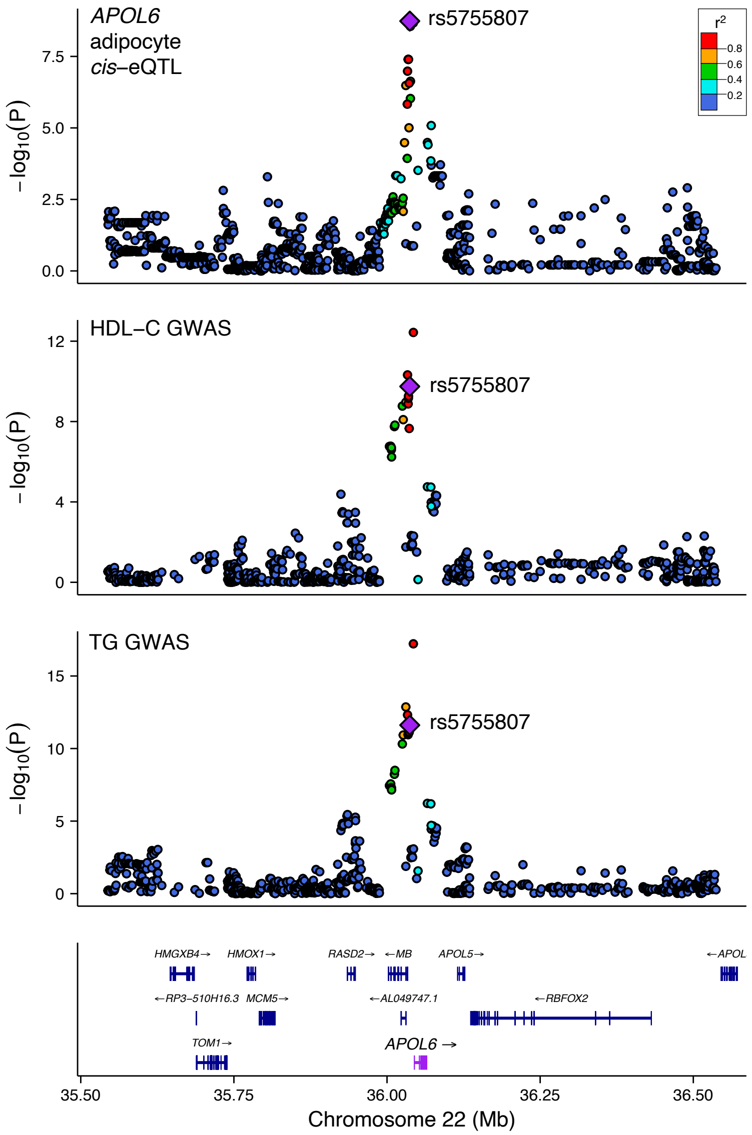

Supplementary Figure 5. Colocalization of the *APOL6 cis*-eQTL variants are unique to adipocytes. LocusZoom plots of adipocyte cell *cis*-eQTL variants for *APOL6*, high-density lipoprotein-cholesterol (HDL-C) GWAS, and triglyceride (TG) GWAS. The lead *cis*-eQTL variant, rs5755807, is indicated by a purple diamond. Colors represent linkage disequilibrium (LD), r^2, with the lead *cis*-eQTL variant. The unadjusted p-values of the *cis*-eQTL variants were computed using two-sided tests as implemented in tensorQTL[31]. The unadjusted p-values of the GWAS variants were derived from the corresponding previously published GWAS studies, listed

in Supplementary Data 12. *APOL6* indicates Apolipoprotein L6; GWAS, genome-wide association study; eQTL, expression quantitative trait loci; Mb, megabase; and P, p-value.

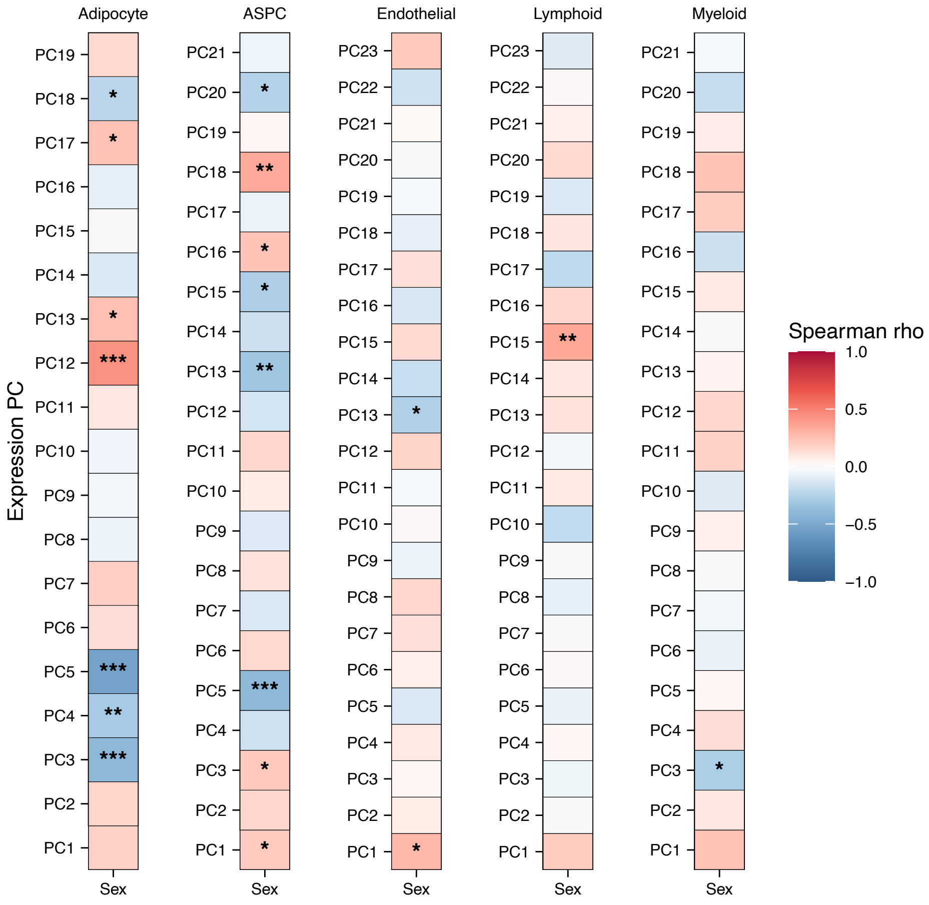

Supplementary Figure 6. Principal components of cell-type level gene expression are correlated with sex. Heatmap showing correlations between sex and top principal components (PCs) computed from the pseudobulk counts of the expressed genes for each cell-type. Asterisks indicate Bonferroni adjusted p-value (*p*.adj) for Spearman correlation (*, *p*.adj < 0.05; **, *p*.adj < 0.01; and ***, *p*.adj < 0.001). ASPC indicates adipose stem and progenitor cells.

Supplementary References

1. Pihlajamäki, J. *et al.* Cholesterol absorption decreases after Roux-en-Y gastric bypass but not after gastric banding. *Metabolism* **59**, 866–872 (2010).
2. Pan, D. Z. *et al.* Identification of TBX15 as an adipose master trans regulator of abdominal obesity genes. *Genome Med.* **13**, 123 (2021).
3. Heinonen, S. *et al.* Roux-en-Y versus one-anastomosis gastric bypass (RYSA study): weight loss, metabolic improvements, and nutrition at 1 year after surgery, a multicenter randomized controlled trial. *Obes. Silver Spring Md* **31**, 2909–2923 (2023).
4. Deal, M. *et al.* An abdominal obesity missense variant in the adipocyte thermogenesis gene TBX15 is implicated in adaptation to cold in Finns. *Am. J. Hum. Genet.* **111**, 2542–2560 (2024).
5. Lee, S. H. T. *et al.* Longitudinal adipose tissue single cell transcriptomics reveals genes and variants regulating weight loss after bariatric surgery. *MedRxiv Prepr. Serv. Health Sci.* 2025.07.11.25331390 (2025) doi:10.1101/2025.07.11.25331390.
6. Chen, Z. J. *et al.* Single-cell DNA methylome and 3D genome atlas of human subcutaneous adipose tissue. *Nat. Genet.* **57**, 2238–2249 (2025).
7. Sudlow, C. *et al.* UK biobank: an open access resource for identifying the causes of a wide range of complex diseases of middle and old age. *PLoS Med.* **12**, e1001779 (2015).
8. Bycroft, C. *et al.* The UK Biobank resource with deep phenotyping and genomic data. *Nature* **562**, 203–209 (2018).
9. Purcell, S. *et al.* PLINK: a tool set for whole-genome association and population-based linkage analyses. *Am. J. Hum. Genet.* **81**, 559–575 (2007).

10. Taliun, D. *et al.* Sequencing of 53,831 diverse genomes from the NHLBI TOPMed Program. *Nature* **590**, 290–299 (2021).
11. Das, S. *et al.* Next-generation genotype imputation service and methods. *Nat. Genet.* **48**, 1284–1287 (2016).
12. Loh, P.-R. *et al.* Reference-based phasing using the Haplotype Reference Consortium panel. *Nat. Genet.* **48**, 1443–1448 (2016).
13. Frankish, A. *et al.* GENCODE reference annotation for the human and mouse genomes. *Nucleic Acids Res.* **47**, D766–D773 (2019).
14. Dobin, A. *et al.* STAR: ultrafast universal RNA-seq aligner. *Bioinformatics* **29**, 15–21 (2013).
15. Alvarez, M. *et al.* Enhancing droplet-based single-nucleus RNA-seq resolution using the semi-supervised machine learning classifier DIEM. *Sci. Rep.* **10**, 11019 (2020).
16. Yang, S. *et al.* Decontamination of ambient RNA in single-cell RNA-seq with DecontX. *Genome Biol.* **21**, 57 (2020).
17. Kang, H. M. *et al.* Multiplexed droplet single-cell RNA-sequencing using natural genetic variation. *Nat. Biotechnol.* **36**, 89–94 (2018).
18. McGinnis, C. S., Murrow, L. M. & Gartner, Z. J. DoubletFinder: Doublet Detection in Single-Cell RNA Sequencing Data Using Artificial Nearest Neighbors. *Cell Syst.* **8**, 329-337.e4 (2019).
19. Hao, Y. *et al.* Integrated analysis of multimodal single-cell data. *Cell* **184**, 3573-3587.e29 (2021).
20. Korsunsky, I. *et al.* Fast, sensitive and accurate integration of single-cell data with Harmony. *Nat. Methods* **16**, 1289–1296 (2019).

21. Aran, D. *et al.* Reference-based analysis of lung single-cell sequencing reveals a transitional profibrotic macrophage. *Nat. Immunol.* **20**, 163–172 (2019).

22. Emont, M. P. *et al.* A single-cell atlas of human and mouse white adipose tissue. *Nature* **603**, 926–933 (2022).

23. Lee, D.-S. *et al.* Simultaneous profiling of 3D genome structure and DNA methylation in single human cells. *Nat. Methods* **16**, 999–1006 (2019).

24. Liu, H. *et al.* DNA methylation atlas of the mouse brain at single-cell resolution. *Nature* **598**, 120–128 (2021).

25. Zhou, J. *et al.* Robust single-cell Hi-C clustering by convolution- and random-walk-based imputation. *Proc. Natl. Acad. Sci. U. S. A.* **116**, 14011–14018 (2019).

26. Schultz, M. D. *et al.* Human body epigenome maps reveal noncanonical DNA methylation variation. *Nature* **523**, 212–216 (2015).

27. He, Y. *et al.* Spatiotemporal DNA methylome dynamics of the developing mouse fetus. *Nature* **583**, 752–759 (2020).

28. Chakraborty, A., Wang, J. G. & Ay, F. dcHiC detects differential compartments across multiple Hi-C datasets. *Nat. Commun.* **13**, 6827 (2022).

29. Iotchkova, V. *et al.* GARFIELD classifies disease-relevant genomic features through integration of functional annotations with association signals. *Nat. Genet.* **51**, 343–353 (2019).

30. Finucane, H. K. *et al.* Partitioning heritability by functional annotation using genome-wide association summary statistics. *Nat. Genet.* **47**, 1228–1235 (2015).

31. Taylor-Weiner, A. *et al.* Scaling computational genomics to millions of individuals with GPUs. *Genome Biol.* **20**, 228 (2019).
