# Supplementary material for: Adipose single cell epigenome and transcriptome localize genetic risk for cardiometabolic disease and accelerated aging
Source: Nat Commun. 2026 Apr 20;17:5469. doi: 10.1038/s41467-026-72248-4 (PMC13284207; doi:10.1038/s41467-026-72248-4)
Supplement: Supplementary file 2 — Description of Additional Supplementary Files [file 41467_2026_72248_MOESM2_ESM.pdf]

Description of Additional Supplementary Files

Supplementary Data 1: Clinical characteristics of the Finnish bariatric surgery subcutaneous adipose tissue (SAT) snRNA-seq cohorts. (Excel)

Supplementary Data 2: Summary statistics of the subcutaneous adipose tissue (SAT) cell-type level lead *cis*-eQTL variants (qval<0.05). Full summary level results are available on Zenodo at https://zenodo.org/records/18880304 (Excel)

Supplementary Data 3. Summary statistics of the conditionally independent subcutaneous adipose tissue (SAT) cell-type level lead *cis*-eQTL variants (qval<0.05). (Excel)

Supplementary Data 4: Replication of the cell-type level primary lead *cis*-eQTL variants in the AdipoExpress (Brotman et al. Nat Genet 2025) SAT bulk RNA-seq meta analysis. (Excel)

Supplementary Data 5: Summary statistics of the subcutaneous adipose tissue (SAT) cell-type level lead *cis*-eQTL variants from SAIGE-QTL (qval<0.05). Full summary level results are available on Zenodo at https://zenodo.org/records/18880304 (Excel)

Supplementary Data 6: Proportion of loss-of-function (LoF) intolerant genes among the cell-type expressed genes are significantly lower for those with *cis*-eQTL variants than those without. (Excel)

Supplementary Data 7: Loss-of-function (LoF) intolerant eGenes show significant enrichment (FDR<0.05) in functional pathway overrepresentation analysis. (Excel)

Supplementary Data 8: Cell-type level primary lead *cis*-eQTL variants are enriched in *cis* regulatory elements and epigenomic features. (Excel)

Supplementary Data 9: Myeloid cell eGenes in A compartments show significant enrichments (FDR<0.05) in functional pathway overrepresentation analysis. (Excel)

Supplementary Data 10: Enrichment of cell-type level primary lead *cis*-eQTL variants in *cis* regulatory elements and epigenomic features in the active (A) and inactive (B) chromatin compartments. (Excel)

Supplementary Data 11: Replication of the cell-type-specific and cell-type level primary lead *cis*-eQTL variants in the GTEx SAT and VAT bulk tissue *cis*-eQTL analyses. (Excel)

Supplementary Data 12: GWAS traits included in the heritability and colocalization analyses. (Excel)

Supplementary Data 13: Heritability analysis of *cis* regional variants of cell-type level eGenes for 36 cardiometabolic and biological aging traits. (Excel)

Supplementary Data 14: Heritability of 36 cardiometabolic and biological aging traits is largely enriched in the *cis* regions of the cell-type level eGenes residing in the active (A) chromatin compartments. (Excel)

Supplementary Data 15: Colocalization results (PP4≥0.5) of the subcutaneous adipose tissue (SAT) cell-type level *cis*-eQTL and 36 cardiometabolic and biological aging trait GWAS variants. (Excel)

Supplementary Data 16: Replication of cell-type level colocalization results (PP4≥0.5) in the AdipoExpress (Brotman et al. Nat Genet 2025) SAT bulk RNA-seq meta analysis for the 25 shared traits between the studies. (Excel)

Supplementary Data 17: Cross-referencing T2D colocalized eGenes against the Drug-Gene Interaction Database (DGIdb) v.5.0.11 reveals interactions with known drugs. (Excel)
