# Supplementary material for: Adipose single cell epigenome and transcriptome localize genetic risk for cardiometabolic disease and accelerated aging
Source: Nat Commun. 2026 Apr 20;17:5469. doi: 10.1038/s41467-026-72248-4 (PMC13284207; doi:10.1038/s41467-026-72248-4)
Supplement: Supplementary file 4 — Reporting Summary [file 41467_2026_72248_MOESM4_ESM.pdf]

nature portfolio

Corresponding author(s): Päivi Pajukanta

Last updated by author(s): Apr 1, 2026

Reporting Summary

Nature Portfolio wishes to improve the reproducibility of the work that we publish. This form provides structure for consistency and transparency in reporting. For further information on Nature Portfolio policies, see our Editorial Policies and the Editorial Policy Checklist.

Statistics

For all statistical analyses, confirm that the following items are present in the figure legend, table legend, main text, or Methods section.

n/a	Confirmed	
☐	☒	The exact sample size (*n*) for each experimental group/condition, given as a discrete number and unit of measurement
☐	☒	A statement on whether measurements were taken from distinct samples or whether the same sample was measured repeatedly
☐	☒	The statistical test(s) used AND whether they are one- or two-sided<br>*Only common tests should be described solely by name; describe more complex techniques in the Methods section.*
☐	☒	A description of all covariates tested
☐	☒	A description of any assumptions or corrections, such as tests of normality and adjustment for multiple comparisons
☐	☒	A full description of the statistical parameters including central tendency (e.g. means) or other basic estimates (e.g. regression coefficient) AND variation (e.g. standard deviation) or associated estimates of uncertainty (e.g. confidence intervals)
☐	☒	For null hypothesis testing, the test statistic (e.g. *F*, *t*, *r*) with confidence intervals, effect sizes, degrees of freedom and *P* value noted<br>*Give P values as exact values whenever suitable.*
☐	☒	For Bayesian analysis, information on the choice of priors and Markov chain Monte Carlo settings
☐	☒	For hierarchical and complex designs, identification of the appropriate level for tests and full reporting of outcomes
☐	☒	Estimates of effect sizes (e.g. Cohen's *d*, Pearson's *r*), indicating how they were calculated

Our web collection on statistics for biologists contains articles on many of the points above.

Software and code

Policy information about availability of computer code

Data collection

No software was used to collect the data in this study.

Data analysis

We combined imputed genotype data from the KOBS and RYSA cohorts and performed quality control for cis-eQTL mapping using PLINK v1.9. All LD-clumping and LD-pruning were also done using PLINK v1.9. Relatedness between individuals was evaluated using KING v2.3.2. The SAT snRNA-seq datasets used in this study were aligned to the GRCh38 human genome referenceand GENCODE v42 annotations using STAR v2.7.10b. We identified the originating individual of each nucleus using demuxlet v2 from popscle software tool and performed quality control on the KOBS and RYSA SAT snRNA-seq datasets using DIEM v2.4.0, Seurat v4.3.0.1, DecontX from celda R package v1.14.2, and DoubletFinder v2.0.3. We combined the two snRNA-seq datasets and performed gene count normalization, variable gene selection, scaling, and PCA on the merged object using Seurat v4.3.0.1. To visualize the integrated datasets, we used Harmony v1.0.3 to perform dimension reduction while accounting for batch effects.

We performed cell-type level cis-eQTL mapping using tensorQTL v1.0.9 (cis_nominal) and SAIGE-QTL v0.3.4. The stepwise regression procedure employed in tensorQTL v1.0.9 (cis_independent) was used to identify conditionally independent secondary and tertiary cis-eQTL signals. We used the multivariate adaptive shrinkage (mash) method v0.2.50 to assess cell-type sharing of cis-eQTL effects. To identify conditionally independent GWAS signals and generate conditional summary statistics we used GCTA v1.94.3. GWASs for biological aging and accelerated biological aging were performed in the UK Biobank using BOLT-LMM v2.3.6.

We tested for enrichments of gene sets in the Gene Ontology biological processes, Reactome pathways, and WikiPathways using WebGestaltR v0.4.6 and enrichments of lead cis-eQTL variants in cis regulatory elements and epigenomic features using GARFIELD v2. We assessed heritability enrichment and depletion of obesity-associated traits using linkage disequilibrium score regression (LDSC v1.0.1). We performed Bayesian colocalization between cell-type level cis-eQTL and GWAS signals using coloc v5.1.0. We constructed polygenic risk scores for T2D

using PLINK v1.9.

The code used in this study is available at https://github.com/seunghyuktlee/SAT_snRNA_eQTL_2026. All codes used for analyses in this study were based on the publicly available source code of the packages listed in the Methods and Supplementary Methods. Additional details are available in the Methods and Supplementary Methods.

For manuscripts utilizing custom algorithms or software that are central to the research but not yet described in published literature, software must be made available to editors and reviewers. We strongly encourage code deposition in a community repository (e.g. GitHub). See the Nature Portfolio guidelines for submitting code & software for further information.

Data

Policy information about availability of data

All manuscripts must include a data availability statement. This statement should provide the following information, where applicable:

- Accession codes, unique identifiers, or web links for publicly available datasets
- A description of any restrictions on data availability
- For clinical datasets or third party data, please ensure that the statement adheres to our policy

The full cell-type level cis-eQTL summary level data, generated using tensorQTL and SAIGE-QTL, and the GWAS summary statistics of the biological aging traits are available in Zenodo [https://zenodo.org/records/18880304]. Previous publications and their links to the summary statistics of the cardiometabolic disease trait GWASs are listed in Supplementary Data 12. The KOBS SAT snRNA-seq data are available in the NIH Gene Expression Omnibus (GEO) under accession number GSE302701 [https://www.ncbi.nlm.nih.gov/geo/query/acc.cgi?acc=GSE302701]. The RYSA SAT snRNA-seq data are available in GEO under accession number GSE274778 [https://www.ncbi.nlm.nih.gov/geo/query/acc.cgi?acc=GSE274778]. The SAT snm3C-seq data are available in GEO under accession number GSE297267 [https://www.ncbi.nlm.nih.gov/geo/query/acc.cgi?acc=GSE297267]. The adipocyte ATAC-seq data are available in GEO, under accession number GSE269929 [https://www.ncbi.nlm.nih.gov/geo/query/acc.cgi?acc=GSE269929]. Data from the UK Biobank were used in this study under UK Biobank Application Number 33934. UK Biobank data are available for bona fide researchers through the application process (https://www.ukbiobank.ac.uk/learn-more-about-uk-biobank/contact-us). Source Data are provided with this paper.

Research involving human participants, their data, or biological material

Policy information about studies with human participants or human data. See also policy information about sex, gender (identity/presentation), and sexual orientation and race, ethnicity and racism.

Reporting on sex and gender	All individuals from the KOBS, RYSA, and Tilkka cohorts provided self-reported sex information and they were cross-checked with the genetically inferred sex from the DNA-level genotype data. Self-reported sex information was used for the UK Biobank cohort. We considered sex in the study design by including it as a covariate where appropriate. We did not perform sex- and gender-based analyses a priori and post hoc sex- and gender-based analysis due to the limited sample size. Additional details are available in the Methods and Discussion.
Reporting on race, ethnicity, or other socially relevant groupings	All study participants are individuals of European ancestry. No socially relevant groupings were considered.
Population characteristics	The KOBS SAT snRNA-seq cohort comprises 59 Finnish participants undergoing bariatric surgery who were recruited in the University of Eastern Finland and Kuopio University Hospital, Kuopio, Finland. Among the 59 participants, 32 are females and 15 have type 2 diabetes (T2D). The mean age of the KOBS snRNA-seq cohort is 49.1 (SD=9.7) with body mass index (BMI) of 40.9 kg/m2 (SD=4.3). The RYSA SAT snRNA-seq cohort comprises 68 Finnish participants undergoing bariatric surgery who were recruited in the Helsinki University Hospital, Helsinki, Finland. Among the 68 participants, 49 are females and 27 have T2D. The mean age of the RYSA snRNA-seq cohort is 46.3 (SD=7.9) with BMI of 43.1 kg/m2 (SD=5.4). Additional details are available in the Supplementary Methods and Supplementary Table 1. The Tilkka SAT snm3C-seq cohort comprises eight Finnish females undergoing abdominal liposuction at Tilkka Hospital, Helsinki, Finland. The mean age of the Tilkka snm3C-seq cohort is 44.4 (SD=4.78). The UK Biobank consists of ~500,000 individuals with a broad range of ages and BMI. We included unrelated individuals of European-origin for the GWAS, heritability, and polygenic risk score analyses and as an LD reference.
Recruitment	Recruitment of the KOBS cohort participants was done at the University of Eastern Finland and Kuopio University Hospital, Kuopio, Finland, and recruitment of the RYSA cohort participants was done at the Helsinki University Hospital, Helsinki, Finland. The Tilkka cohort participants were recruited at Tilkka Hospital, Helsinki, Finland, and the UK Biobank study participants were recruited across 22 assessment centers in UK. Additional details are available in Supplementary Methods.
Ethics oversight	The Kuopio Obesity Surgery Study (KOBS) was approved by the Ethics Committee of the Northern Savo Hospital District (54/2005, 104/2008, and 27/2010). The longitudinal Roux-en-Y versus one-anastomosis gastric bypass (RYSA) study was approved by the Helsinki University Hospital Ethics Committee (HUS/1706/2016). The Tilkka study was approved by the Helsinki University Hospital Ethics Committee (HUS/1039/2019). The UKB study was approved by the North West Multi-centre Research Ethics Committee (21/NW/0157). All participants in these studies provided a written informed consent, and no compensation was provided to the participants. All research was conducted in accordance with the principles of the Declaration of Helsinki.

Note that full information on the approval of the study protocol must also be provided in the manuscript.

Field-specific reporting

Please select the one below that is the best fit for your research. If you are not sure, read the appropriate sections before making your selection.

☒ Life sciences ☐ Behavioural & social sciences ☐ Ecological, evolutionary & environmental sciences

For a reference copy of the document with all sections, see nature.com/documents/nr-reporting-summary-flat.pdf

Life sciences study design

All studies must disclose on these points even when the disclosure is negative.

Sample size

No statistical method was used to predetermine the sample size. The KOBS and RYSA cohorts comprises 59 and 68 SAT snRNA-seq samples, respectively, for a total of 127 samples for the cis-eQTL analysis. Our sample size is comparable to those reported in previous cell-type level cis-eQTL study in lung tissue (Natri et al. Nat Genet. 2024). The sample size reflects the availability of both genotype data and SAT biopsies.

Data exclusions

To ensure the statistical validity of the cis-eQTL mapping, we had pre-established a criterion to exclude related individuals. We had confirmed that no individuals among the two SAT snRNA-seq cohorts are related up to 3rd degree. Thus, no individuals or their data were excluded from our study. This information is also available in the Methods section.

Replication

We evaluated replication rates of our cell-type level lead cis-eQTL variants and colocalized cis-eQTL GWAS variant pairs in the large SAT bulk meta-analysis (Brotman et al. Nat Genet. 2025). We observed that 54–78% of our cell-type level cis-eQTL variants and 33% of our colocalized cis-eQTL and GWAS variant pairs replicated in the bulk study. However, the results of the two studies are not fully comparable due to the difference in single cell and bulk tissue RNA-seq modalities, and thus the resolution difference between the two studies. Furthermore, our study comprises individuals with obesity while the bulk study is limited to relatively healthy individuals. Due to the unique nature of our SAT snRNA-seq data from individuals with obesity and the lack of other large SAT snRNA-seq cohorts, our results cannot directly be replicated using the same modality. The results from the UK Biobank data were not replicated due to the unprecedentedly large sample size of the cohort. Additional details are available in Methods.

Randomization

N/A. This is an observational study, so no randomization was performed.

Blinding

N/A. This is an observational study, so no blinding was performed.

Reporting for specific materials, systems and methods

We require information from authors about some types of materials, experimental systems and methods used in many studies. Here, indicate whether each material, system or method listed is relevant to your study. If you are not sure if a list item applies to your research, read the appropriate section before selecting a response.

Materials & experimental systems

n/a	Involved in the study
☒	☐ Antibodies
☒	☐ Eukaryotic cell lines
☒	☐ Palaeontology and archaeology
☒	☐ Animals and other organisms
☒	☐ Clinical data
☒	☐ Dual use research of concern
☒	☐ Plants

Methods

n/a	Involved in the study
☒	☐ ChIP-seq
☒	☐ Flow cytometry
☒	☐ MRI-based neuroimaging

Plants

Seed stocks

Report on the source of all seed stocks or other plant material used. If applicable, state the seed stock centre and catalogue number. If plant specimens were collected from the field, describe the collection location, date and sampling procedures.

Novel plant genotypes

Describe the methods by which all novel plant genotypes were produced. This includes those generated by transgenic approaches, gene editing, chemical/radiation-based mutagenesis and hybridization. For transgenic lines, describe the transformation method, the number of independent lines analyzed and the generation upon which experiments were performed. For gene-edited lines, describe the editor used, the endogenous sequence targeted for editing, the targeting guide RNA sequence (if applicable) and how the editor was applied.

Authentication

Describe any authentication procedures for each seed stock used or novel genotype generated. Describe any experiments used to assess the effect of a mutation and, where applicable, how potential secondary effects (e.g. second site T-DNA insertions, mosiacism, off-target gene editing) were examined.
