# Supplementary material for: Adipose single cell epigenome and transcriptome localize genetic risk for cardiometabolic disease and accelerated aging
Source: Nat Commun. 2026 Apr 20;17:5469. doi: 10.1038/s41467-026-72248-4 (PMC13284207; doi:10.1038/s41467-026-72248-4)
Supplement: Supplementary file 5 — Transparent Peer Review file [file 41467_2026_72248_MOESM5_ESM.pdf]

nature portfolio

Peer Review File

Adipose single cell epigenome and transcriptome localize genetic risk for cardiometabolic disease and accelerated aging

Corresponding Author: Professor Päivi Pajukanta

This file contains all reviewer reports in order by version, followed by all author rebuttals in order by version.

Version 0:

Reviewer comments:

Reviewer #1

(Remarks to the Author)
The authors investigated regulatory variants and target genes in obesity that predispose individuals to CMD and accelerated aging. By integrating single cell adipose cis eQTL mapping, enrichment analysis using 3D chromatin profiles, and colocalization with GWAS data, they demonstrate that cell type-specific approaches reveal more cis-eQTLs than bulk tissue studies, particularly in adipocytes. These cis-eQTLs and eGenes show strong cell-type specificity and enrichment in active chromatin compartments, with heritability analyses highlighting their relevance to disease traits. Colocalization analyses further identify numerous adipocyte-specific variants and genes, underscoring the role of 3D chromatin architecture in disease regulation. The prioritized variants and genes perform well in predicting T2D status, pointing to their functional and translational significance.

MAJOR COMMENTS

1. The abstract claims that mechanisms are uncovered, but the results section does not fully elaborate this point. Consider adding further analyses such as TF binding motif enrichment in cis regions of colocalized eGenes, or trans eQTL analyses, to strengthen mechanistic insights.

2. A deeper exploration of the 41 colocalized eGenes used in T2D prediction would strengthen the manuscript. For example, pathway analysis, assessment of biological relevance, and cross referencing with drug repositories or gene databases would add value.

3. Provide a clearer description of the snm3C seq data, including how active and inactive compartments are defined, to aid readers unfamiliar with the method.

4. Please justify the choice of UMI threshold (500 vs. 400 in e.g. Emont et al. Nature 2022) and consider whether increasing the PP4 threshold from 0.5 to 0.8 would provide more robust evidence for colocalization.

5. Consider emerging single cell eQTL tools (e.g., tensorQTL, SAIGE QTL) that address limitations of pseudobulk approaches and linear regression models.

6. For ROADMAP samples, beyond E063, consider including adipocyte sample E023 (as in e.g. Dong et al. Am J Hum Genet 2025). Please specify whether you used the 15 state model for annotations.

7. The manuscript notes the focus on five cell types and the absence of healthy individuals. Please elaborate how these limitations might affect generalizability.

8. In enrichment analyses described in Supplementary Tables 6 and 8, consider including additional pathway resources (Reactome, WikiPathways, MSigDB) and exploring lymphoid cell results.

9. Was the partitioned T2D PRS that showed interaction with BMI also directly associated with BMI? If so, this would

complicate interpretation of the observed interaction, as part of the apparent effect could reflect underlying correlation between the PRS and BMI rather than a true synergistic effect.

MINOR COMMENTS

1. In the Abstract, consider revising “These findings uncover mechanisms for…” to better align with the depth of mechanistic results presented.

2. Enrichment of adipocyte promoters was also found in inactive (B) compartments, as shown in Fig 1h and Supplementary Table 9. Please clarify the biological relevance of enrichment in inactive compartments. Also revise the title of Supplementary Table 9 for accuracy.

3. Figures 3 and 4 reference Supplementary Table 10 for heritability and colocalization results, but these are actually in ST11–13. Please correct these references.

Reviewer #2

(Remarks to the Author)
I co-reviewed this manuscript with one of the reviewers who provided the listed reports. This is part of the Nature Communications initiative to facilitate training in peer review and to provide appropriate recognition for Early Career Researchers who co-review manuscripts.

Reviewer #3

(Remarks to the Author)
The manuscript titled "Adipose single cell epigenome and transcriptome localize genetic risk for cardiometabolic disease and accelerated aging" presents an impressive and timely study that tackles the complexity of obesity-associated diseases by dissecting the subcutaneous adipose tissue (SAT) at single-cell resolution.
The integration of snRNA-seq with snm3C-seq to map the 3D epigenomic landscape is particularly commendable. The authors' finding that the active "A" compartments in adipocytes serve as a hub for disease heritability provides a compelling structural mechanism for how genetic risk is translated into phenotype. Furthermore, the identification of cell-type-specific cis-eQTLs that were missed by previous bulk studies highlights the unique value of the dataset.
Overall, the experimental design is rigorous, and the manuscript is well-written. A few areas have been identified where additional computational checks and clarifications could further strengthen the authors' conclusions and broaden the impact of the work.

Major Comments
1. Validating the Predominance of Adipocyte eGenes: A key finding is that adipocytes harbor the vast majority of detected eGenes (1,215 out of 1,684). While this aligns with the biological role of adipocytes in SAT, Figure 1 indicates a correlation between the number of nuclei sequenced and the number of eGenes found. Although the authors utilized a modified Z-score to correct for this, a direct empirical validation would be very persuasive.
Suggestion: To definitively rule out sample size bias, it is recommended that the authors perform a down-sampling analysis. The authors should randomly subsample the adipocyte nuclei to match the counts of less abundant cell types (e.g., endothelial or lymphoid cells) and re-run the eQTL mapping. If adipocytes still show a higher number (or higher density) of eGenes compared to other cell types at the same sequencing depth, this will strongly solidify the conclusion that adipocytes are the primary regulatory hub.

2. Generalizability of the 3D Epigenome Map: The snm3C-seq data provides a unique view of the chromatin architecture. As noted, this data is derived from 5 female donors. While this is a standard sample size for such a high-complexity assay, discussing its representativeness would be beneficial for the reader.
The authors are advised to add a discussion paragraph or a supplementary analysis addressing the generalizability of the A/B compartment maps. If feasible, comparing the compartment calls with publicly available bulk Hi-C data from human adipose tissue would serve as an excellent "sanity check" to demonstrate that the broad structural features are consistent with the larger population. This would reassure readers that the N=5 dataset is a reliable reference for the broader N=127 cohort.

3. Robustness of the T2D Prediction Model: The Elastic Net model predicting T2D status using 41 colocalized eGenes is a promising clinical application (AUC 0.74). Given the cohort size (N=127), a single 70:30 train/test split can sometimes be sensitive to how the data is partitioned.
To demonstrate the stability of the model, the authors should consider using a Leave-One-Out Cross-Validation (LOOCV) or a repeated k-fold cross-validation approach (e.g., 50 or 100 iterations). Reporting the mean AUC and its standard deviation from these iterations will provide a more robust estimate of the model's performance and protect against potential overfitting.

4. Clarification of Interaction Analysis (Figure 6b): The finding that the partitioned PRS interacts with BMI to increase T2D risk is highly significant. However, it is not immediately clear in the text whether this specific interaction analysis was powered by the local bariatric cohort (N=127) or if it was conducted using the UK Biobank dataset.
The authors should clarify the data source for this specific analysis in the main text and figure caption. If it was performed in the local cohort, a brief mention of the statistical power or a caveat regarding sample size for interaction terms would be

appropriate to contextualize the findings.

Minor Comments
1. Methodological Detail: The methods section for snm3C-seq is quite concise. Expanding the Supplementary Methods to include more details on library preparation, sequencing depth per nucleus, and QC metrics would greatly assist the community in reproducing this cutting-edge technique.

2. Terminology: The terms "sn-eQTL" (single-nucleus) and "sc-eQTL" (single-cell) appear occasionally. For consistency, it is recommended that the authors stick to one term (likely "sn-eQTL" given the methods) throughout the manuscript.

3. Data Availability: The authors should ensure that the summary statistics for the cell-type-specific eQTLs are made easily accessible (e.g., via a repository or supplementary table). This will be a valuable resource for future meta-analyses.

4. Figure 3 and Supplementary Figure 2 appear to visualize the same heritability enrichment results, but the x-axis scales differ noticeably between the two figures. This makes it difficult to directly compare effect magnitudes across the main and supplementary panels. For clarity and consistency, it would be helpful to harmonize the axis ranges or explain the rationale for using different scales.

5. The manuscript and Figure 5a reports that 90% of colocalized eGenes are cell type–specific, while the mashr analysis shows that only 30–56% of the eGenes are cell type–specific. I understood these figures reflect different levels of specificity —variant-level colocalization versus gene-level eQTL sharing— but this may be confusing to readers. Clarifying the distinction between these two concepts would improve interpretability and coherence of the results.

Reviewer #4

(Remarks to the Author)
This manuscript by Lee et al. integrates single-nucleus RNA-seq and single-cell epigenomic data from subcutaneous adipose tissue (SAT) of individuals with obesity to map cell-type-specific cis-eQTLs and link them to cardiometabolic disease (CMD) and aging traits. The authors identify 1,684 eGenes, discover 682 colocalized eQTL-GWAS pairs, and show enrichment of colocalized variants in active chromatin compartments. They also demonstrate predictive utility for T2D using gene expression and a partitioned polygenic risk score (PRS). While the study is technically rigorous, the relatively small sample size (n=127) limits detection of eQTLs in rarer cell types and the obesity-only context may to some extent limit generalizability. Some additional analyses could further increase the impact and resource value of this work.

Major comments:

1. The manuscript should better articulate what is fundamentally new beyond previous SAT bulk eQTL studies (e.g., Brotman et al., Nat Genet 2025).
2. It is unclear whether sex was included as a covariate in all analyses. According to SAGER guidelines, please clarify and justify why sex-stratified analyses were not feasible.
3. The current approach (coloc) to identify shared causal variants between cell-type-specific cis-eQTLs and GWAS signals assumes a single causal variant per locus, which may oversimplify complex loci. To strengthen confidence in the colocalization analyses, key findings should be validated using an alternative method that accommodates multiple causal variants.
4. To increase the ressource value of the manuscript, the authors might compare SAT cell-type-specific eQTLs with visceral adipose tissue (VAT) or GTEx adipose depots to assess tissue specificity.
5. The statement on code availability is insufficient for reproducibility. While the authors indicate that 'no custom code was used', the complexity of the analyses suggests that custom scripts or workflows were necessary. Please provide access to all analysis scripts, pipelines, and parameter settings used in this study via a public repository, with initial access for reviewers. This is essential for assessing the reproducibility of the study.

Version 1:

Reviewer comments:

Reviewer #1

(Remarks to the Author)
The authors have thoroughly addressed all comments. We now recommend the manuscript for acceptance.

Reviewer #2

(Remarks to the Author)
I co-reviewed this manuscript with one of the reviewers who provided the listed reports. This is part of the Nature Communications initiative to facilitate training in peer review and to provide appropriate recognition for Early Career Researchers who co-review manuscripts.

Reviewer #3

(Remarks to the Author)
I commend the authors for their thorough, thoughtful, and transparent responses to my comments and those of the other reviewers.

I particularly appreciate the rigorous approach taken to address the methodological concerns:

1. Down-sampling Analysis: The down-sampling analysis provides compelling empirical evidence supporting the predominance of adipocyte eGenes, significantly strengthening your primary conclusion.

2. T2D Prediction Model & Interaction Analysis: I respect the authors' scientific integrity in rigorously re-evaluating the T2D prediction model (via repeated k-fold CV) and the interaction analysis (by accounting for the underlying association between the partitioned T2D PRS and BMI). Removing these unstable/confounded results and reframing the discussion around polygenic correlations was the correct and most scientifically sound decision.

The manuscript is now much stronger, more rigorous, and its conclusions are well-supported by the data. I have no further concerns and recommend the manuscript for publication.

Reviewer #4

(Remarks to the Author)
The authors have adequately addressed my concerns.

Open Access This Peer Review File is licensed under a Creative Commons Attribution 4.0 International License, which permits use, sharing, adaptation, distribution and reproduction in any medium or format, as long as you give appropriate credit to the original author(s) and the source, provide a link to the Creative Commons license, and indicate if changes were made.
In cases where reviewers are anonymous, credit should be given to 'Anonymous Referee' and the source.
The images or other third party material in this Peer Review File are included in the article’s Creative Commons license, unless indicated otherwise in a credit line to the material. If material is not included in the article’s Creative Commons license and your intended use is not permitted by statutory regulation or exceeds the permitted use, you will need to obtain permission directly from the copyright holder.
To view a copy of this license, visit https://creativecommons.org/licenses/by/4.0/

Responses to the Reviewers

Responses to Reviewer #1
We thank the Reviewer for the helpful comments and suggestions on the manuscript. We have carefully revised the manuscript to address all of the Reviewer's comments. On behalf of the authors, we believe that the manuscript has been substantially improved as a result of these insightful comments. The numbering of the revised pages we provide below refers to the tracked changes version of the revised manuscript. We also provide a clean manuscript. In our responses, we refer to previous papers using their PubMed IDs.

Reviewer #1 (Remarks to the Author)

The authors investigated regulatory variants and target genes in obesity that predispose individuals to CMD and accelerated aging. By integrating single cell adipose cis eQTL mapping, enrichment analysis using 3D chromatin profiles, and colocalization with GWAS data, they demonstrate that cell type-specific approaches reveal more cis-eQTLs than bulk tissue studies, particularly in adipocytes. These cis-eQTLs and eGenes show strong cell-type specificity and enrichment in active chromatin compartments, with heritability analyses highlighting their relevance to disease traits. Colocalization analyses further identify numerous adipocyte-specific variants and genes, underscoring the role of 3D chromatin architecture in disease regulation. The prioritized variants and genes perform well in predicting T2D status, pointing to their functional and translational significance.

Response to the Reviewer's general comment:
We thank the Reviewer for the helpful and constructive comments. We have now carefully revised the manuscript to address all of the Reviewer's comments. These include multiple additional computational analyses detailed below, targeted to further explore genes and their underlying biological mechanisms. As suggested by the Reviewer, we have also performed *cis*-eQTL mapping using SAIGE-QTL (PMID: 38798318), a single cell eQTL tool, and provide the full *cis*-eQTL summary results from SAIGE-QTL as an additional resource for the scientific community. Overall, we are very grateful for the Reviewer's thorough feedback as we feel that these revisions have substantially strengthened and clarified the main findings of our study.

MAJOR COMMENTS

Comment #1. The abstract claims that mechanisms are uncovered, but the results section does not fully elaborate this point. Consider adding further analyses such as TF binding motif enrichment in cis regions of colocalized eGenes, or trans eQTL analyses, to strengthen mechanistic insights.

Response #1. We thank the Reviewer for this insightful comment. As our sample size does not allow adequately powered *trans*-eQTL analysis, we have now performed a TF binding motif enrichment analysis for the *cis* regions of all colocalized eGenes using HOMER (PMID: 20513432). We observed two significantly (p-value=1×10^{-12}) enriched motifs, for AT-rich

interactive domain-containing protein 5a (Arid5a) and highly divergent homeobox (Hdx). Previous mouse studies have shown that Arid5a regulates adipogenesis through a counter-regulation with Ppar-γ2 and that Arid5a deficiency in mice leads to adult-onset obesity (PMID: 31289228). In addition, Arid5a is known to promote inflammation through stabilizing proinflammatory mRNAs (PMIDS: 29358370, 32035762, 35126382). Overall, the enrichment of the Arid5a binding motif in the *cis* regions of colocalized eGenes suggests transcriptional regulation by Arid5a and downstream effectors of Arid5a-mediated adipogenesis and inflammatory regulations as one of the potential biological mechanisms underlying cardiometabolic and biological aging risks under obesity. We have now added these new results into the revised Results (pages 14 and 15) and Methods (page 41).

Comment #2. A deeper exploration of the 41 colocalized eGenes used in T2D prediction would strengthen the manuscript. For example, pathway analysis, assessment of biological relevance, and cross referencing with drug repositories or gene databases would add value.

Response #2. We thank the Reviewer for this constructive comment. We have now further explored the eGenes used in the T2D prediction by assessing their biological relevance based on literature search and cross-referencing against the Drug-Gene Interaction Database (DGIdb) v.5.0.11 (PMID: 37953380). Please note that among the 41 cell-type level eGenes, there are 35 unique genes across the five cell-types. We observed that seven out of the 35 unique eGenes (20%) are known to interact with drugs. Among these seven genes, there is an adipocyte eGene *MARK3*. A previous study has shown that the siRNA-mediated knockdown of *Mark3* in mouse-derived 3T3-L1 adipocytes increases insulin-stimulated GLUT4 translocation, resulting in insulin hypersensitivity (PMID: 36808134). Thus, the agents targeting MARK3 may have a therapeutic potential for T2D upon further investigation.

Some of the notable drugs from the analysis include cilostazol and pentoxifylline. These are inhibitors of phosphodiesterase, such as phosphodiesterase 3A, which is encoded by an adipose stem and progenitor cell (ASPC) eGene, *PDE3A*, and known to hydrolyze cAMP (PMID: 40497949). In mouse-derived 3T3-L1 preadipocytes, cAMP contributes to an increased transcription of *Pparg*, a master regulator of adipogenesis, through activation of protein kinase A (PKA) (PMID: 20638365). Thus, inhibition of PDE3A may contribute to increase in preadipocyte differentiation. In line with this, cilostazol has been shown to promote differentiation of 3T3-L1 preadipocytes to adipocytes via activation of PPARG and increase insulin sensitivity (PMID: 18355828). Similarly, reduction in insulin resistance was observed with pentoxifylline therapy in a previous randomized study (PMID: 26300986).

Interestingly, several second-generation antipsychotics including amisulpride, aripiprazole lauroxil, clozapine, olanzapine, paliperidone, quetiapine fumarate, and risperidone are shown to interact with *SH2B1*, an adipocyte eGene that encodes SH2B adaptor protein 1. We discussed in the previous version of the manuscript that knockout of *Sh2b1* in mice adipose tissue results in an impaired insulin signaling, followed by severe hyperglycemia, hyperinsulinemia, and glucose intolerance (page 26). Second-generation antipsychotics are reported to have adverse effects of increased bodyweight and increased risk of diabetes, hyperglycemia, and dyslipidemia as well as new-onset diabetes in the absence of obesity (PMID: 15998156, 39350614). These converging findings suggest that the interaction between SH2B1 and second-generation antipsychotic in adipose tissue may contribute to the observed T2D risk. Overall, cross referencing our colocalized eGenes against the drug database provides orthogonal

support for the role of several of the colocalized eGenes in T2D risk and highlights therapeutic potential of these genes. We have now added these findings to the revised Results (page 21), Discussion (page 25), and Methods (page 44).

Comment #3. Provide a clearer description of the snm3C seq data, including how active and inactive compartments are defined, to aid readers unfamiliar with the method.

Response #3. We thank the Reviewer for this thoughtful comment. As suggested by the Reviewer, we have now revised the Supplementary Methods (pages 5–8) to provide a more detailed description of the snm3C seq data, including how active and inactive compartments were defined.

Comment #4. Please justify the choice of UMI threshold (500 vs. 400 in e.g. Emont et al. Nature 2022) and consider whether increasing the PP4 threshold from 0.5 to 0.8 would provide more robust evidence for colocalization.

Response #4. We thank the Reviewer for this helpful comment. The UMI threshold of 500 was used in the previous studies of these RYSA snRNA-seq data (PMIDs: 39515300, 40791725), and thus we did not change it here. This cut-point was originally selected based on the UMI distribution plots across all nuclei in our data. We would like to note that UMI thresholds in sc/snRNA-seq are highly data-specific and dependent on technical factors, such as tissue type, cell/nuclei isolation protocol, library construction kit, and sequencing depth. Thus, we empirically selected the UMI threshold of 500 specifically in our data that would ensure high-quality nuclei while reducing empty and ambient RNA droplets. We would like to also note that 99% of the nuclei in the previous human subcutaneous adipose tissue (SAT) snRNA-seq data, an atlas resource provided by Emont et al. (PMID: 35296864), have UMIs of >500. Overall, we consider that a difference in the UMI thresholds would unlikely largely change the downstream results while the used threshold keeps our results in line with our and others' previous work. We have now clarified our rationale for this cut-point in the revised Supplementary Methods (page 4).

We used the PP4 threshold of 0.5 to define evidence of colocalization to be consistent with the threshold used in the SAT bulk *cis*-eQTL meta-analysis and colocalization study (PMID: 39747594), to which we directly compared our colocalization results. Using the same threshold ensures methodological comparability between the two studies. Importantly, we would like to highlight that we provide the full colocalization results (PP4>0.5) along with their corresponding PP4 values. Thus, we ensure transparency while allowing the readers to independently apply more stringent PP4 thresholds based on their preference to evaluate evidence of colocalization. We have now clarified our rationale for this cut-point in the revised Methods (page 41).

Comment #5. Consider emerging single cell eQTL tools (e.g., tensorQTL, SAIGE QTL) that address limitations of pseudobulk approaches and linear regression models.

Response #5. We thank the Reviewer for this constructive comment. As suggested by the Reviewer, we have now performed cell-type level *cis*-eQTL mapping using SAIGE-QTL (PMID: 38798318) for the five SAT cell-types presented in this study. We discovered 1,340 eGenes in adipocytes, 295 in ASPCs, 289 in endothelial cells, 133 in lymphoid cells, and 560 in myeloid

cells, respectively. These eGenes include 74–78% of the eGenes per cell-type that were discovered with pseudobulk mean expression using tensorQTL, demonstrating a high degree of concordance between the two eQTL approaches. This result suggests that the cell-type level *cis*-eQTLs discovered in this study are robust and reproducible across different methodological frameworks, especially considering the differences in the underlying statistical models (linear regression vs Poisson mixed model) and *p*-value estimation procedures implemented by tensorQTL and SAIGE-QTL. We have now added the *cis*-eQTL results generated using SAIGE-QTL into the revised Results (page 7), Supplementary Table 5, and Methods (page 33). Additionally, to increase the resource value of this study for the scientific community, we have now deposited the summary statistics of the *cis*-eQTLs from SAIGE-QTL for each cell-type on Zenodo at https://zenodo.org/records/18880304 and will publicly open the access to these data upon acceptance of the manuscript. We have also revised the Data availability statement accordingly (page 45). The data deposited on Zenodo can be accessed by the reviewers using the following private link:
https://zenodo.org/records/18880304?preview=1&token=eyJhbGciOiJIUzUxMiJ9.eyJpZCI6IjYxOTA2YTJkLTY4YzEtNGI1YS1hYmFkLWJjN2UxMWM4NzlkOCIsImRhdGEiOnt9LCJyYW5kb20iOiIzZDcyZDg2ZDViYzkxODg4NDQ5OGM0ZjZlNjQ1YTc3MiJ9.2IUgNYKuCMxC_P5Dc6w21z1xG_UQ1uZ8uIBeMpBEOxs6Ur5SH95sJMtT9iBWor5FyD4ilo9jVQo-TFlsfaZOXQ

Comment #6. For ROADMAP samples, beyond E063, consider including adipocyte sample E023 (as in e.g. Dong et al. Am J Hum Genet 2025). Please specify whether you used the 15 state model for annotations.

Response #6. We thank the Reviewer for this helpful suggestion. We had included only annotations from the adipose tissue sample E063 to reduce the burden of multiple testing since the annotations from the adipocyte sample E023 would be pertinent to only adipocytes. However, as suggested by the Reviewer, we have now included the annotations from the adipocyte sample E023 for the enrichment of adipocyte *cis*-eQTL variants as well. Overall, adipocyte *cis*-eQTL variants residing in the A chromatin compartment are significantly enriched for enhancers and enhancers from both E063 and E023 samples and the variants in the B chromatin compartment are significantly enriched for promoters from both E063 and E023 samples. All other significant enrichments remained the same. For the Roadmap sample annotations, we used the 25-state model. We have now added the enrichment results with the annotations from the E023 sample to the revised Results (page 8–10), Figure 1h, Supplementary Fig. 1d, Supplementary Tables 8 and 10 as well as clarified the model used for annotations in the revised Methods (page 34).

Comment #7. The manuscript notes the focus on five cell types and the absence of healthy individuals. Please elaborate how these limitations might affect generalizability.

Response #7. We thank the Reviewer for this helpful comment. We focused on the five most abundant cell-types to increase our power to map *cis*-eQTLs. However, we acknowledge that this design limits discovery of regulatory effects that are specific to rare cell-types, which may have important biological role in adipose tissue under obesity. Furthermore, it is possible that the *cis*-eQTLs discovered in this study may not fully capture the full gene regulatory effects present in the SAT. Nevertheless, the five cell-types selected for the main analysis represent the major

cellular lineages present in SAT. Moreover, as part of our response to Reviewer #3's comment #1, we now show that adipocytes have greater number of eGenes discovered even after down-sampling the number of nuclei to match that of less abundant cell-types, endothelial and lymphoid cells. This result from the down-sampling analysis further supports potential increase in regulation of adipocytes under obesity. Taken together, the *cis*-eQTLs discovered in this study likely comprise the core genetic regulatory components of the SAT under obesity, while future studies with larger sample size would strengthen complete genetic regulatory mapping of SAT. We have now discussed this limitation in the revised Discussion (page 26).

The absence of healthy individuals in our study cohort limits generalizability of our findings to entire population as the regulatory effects discovered in individuals with obesity may not fully represent the baseline gene regulations in healthy individuals. Additionally, certain *cis*-eQTL signals may be specific or dependent under the context of obesity, with effect sizes being amplified or reduced. However, given that previous SAT bulk tissue studies (PMIDs: 28257690, 31564431, 39747594) are largely limited to healthy individuals, there is a value in characterizing genetic regulations within the context of obesity to better understanding the underlying disease biology. It is worth noting that our data are still highly relevant for the large proportion of the population as the individuals with obesity (BMI≥30) comprise ~40% of adults in the US, with similar rates reported in an increasing number of countries world-wide (PMID: 39808758). Furthermore, the *cis*-eQTLs discovered in our study cohort likely also include many of those that are broadly shared across conditions. Future SAT cell-type-level *cis*-eQTL studies comprising healthy individuals would allow for assessment of disease-shared vs specific regulatory effects. We have now further discussed this limitation in the revised Discussion (page 26).

Comment #8. In enrichment analyses described in Supplementary Tables 6 and 8, consider including additional pathway resources (Reactome, WikiPathways, MSigDB) and exploring lymphoid cell results.

Response #8. We thank the Reviewer for this helpful suggestion. We have now included Reactome and WikiPathways databases for the pathway enrichment analyses, described in Supplementary Tables 7 and 9 (previously Supplementary Tables 6 and 8). For the myeloid cell eGenes in A compartments (Supplementary Table 9), we observed one additional pathway, "Immune System", from the Reactome database. This result is in line with the Gene Ontology terms enriched by these eGenes. No additional enrichments were observed with eGenes residing within the other cell-type level A compartments. We did not observe significant pathway enrichments with any lymphoid cell eGenes, potentially due to the smaller number of eGenes discovered in lymphoid cells than in other cell-types.

For the loss-of-function (LoF) intolerant eGenes (Supplementary Table 7), we observed overrepresentation of one pathway from Reactome database, i.e., signal transduction for myeloid cells, suggesting that regulation of these genes constrains core signal architecture in myeloid cells. In addition, we observed ten pathways from Reactome database and five pathways from Wikipathway database for adipocytes. These pathways include mitogen-activated protein kinase (MAPK) family signaling cascades and deubiquitination, well-established regulators of adipocyte differentiation (PMID: 15733737, 24141283). Another example is the Human Thyroid Stimulating Hormone (TSH) signaling pathway, which is involved in regulation of lipolysis (PMID: 19846175). These examples show that genetic regulation of the genes in these pathways may modify adipocyte differentiation and lipolysis, which contributes to adipose tissue

dysfunction and increased risk for obesity and metabolic complications. We have now included these updated pathway enrichment analyses and examples in the revised Results (pages 8 and 9), Methods (pages 34 and 36), and Supplementary Tables 7 and 9.

Comment #9. Was the partitioned T2D PRS that showed interaction with BMI also directly associated with BMI? If so, this would complicate interpretation of the observed interaction, as part of the apparent effect could reflect underlying correlation between the PRS and BMI rather than a true synergistic effect.

Response #9. We thank the Reviewer for this very helpful comment that helped us clarify our partitioned T2D PRS interaction result. As the Reviewer suggested, we have now tested for the association between the partitioned T2D PRS and BMI. We observed a significant positive association (beta=0.02, p-value=5.35×10^{-45}), and thus the partitioned T2D PRS predicts both T2D and BMI. This result also suggests that the observed interaction could be reflecting the underlying correlation between the T2D PRS and BMI, as indicated by the Reviewer. To further dissect these results, we first residualized BMI on the partitioned T2D PRS and covariates to remove the component of BMI that is driven by the T2D PRS. When we then retested for the interaction between this residualized BMI and the partitioned T2D PRS, we observed that the interaction effect is not significant anymore (p-value>0.05) while the main effect of the T2D PRS is significant (beta=0.13, p-value=1.36×10^{-40}). This supports the conclusion that the originally observed interaction effect between the partitioned T2D PRS and BMI may be largely due to the underlying association between the partitioned T2D PRS and BMI. To corroborate the genetic effect on T2D at the colocalized loci, we also reexamine the main effect of the partitioned T2D PRS on T2D after accounting for BMI. We discovered that the partitioned T2D PRS is still a significant predictor of T2D (beta=0.11, p-value=6.10×10^{-44}), suggesting that the polygenic effects on T2D are not fully explained by BMI. Overall, these results demonstrate complex genetic correlations between the partitioned T2D PRS and both T2D and BMI and reveal polygenic correlations, where the underlying functions and pathways of the colocalized T2D genes also likely have pleiotropic effects on obesity. These new results are in line with the fact that the colocalized loci were discovered using eQTLs from individuals with obesity and that obesity is a main risk factor for T2D. Accordingly, we have now revised the Results (page 20) and Methods (pages 43 and 44) to show these partitioned polygenic correlations while also removing Figure 6b. In more detail, we have now replaced all main T2D PRS results with the model that includes BMI as a covariate; added the association between the partitioned T2D PRS and BMI; discussed the potential implications underlying the association, as described above; and deleted the previous partitioned T2D PRS and BMI interaction result.

MINOR COMMENTS

Comment #1. In the Abstract, consider revising “These findings uncover mechanisms for…” to better align with the depth of mechanistic results presented.

Response #1. We thank the Reviewer for this helpful suggestion. We have now revised the sentence in the Abstract (page 2).

Comment #2. Enrichment of adipocyte promoters was also found in inactive (B) compartments, as shown in Fig 1h and Supplementary Table 9. Please clarify the biological relevance of enrichment in inactive compartments. Also revise the title of Supplementary Table 9 for accuracy.

Response #2. We thank the Reviewer for this constructive comment. The enrichment for promoters by the adipocyte eGenes residing in inactive (B) compartments suggests that while the overall chromatin region is inactive in B compartments, genetic regulations of local promoters stays present. These promoters may reflect a poised state with regulatory potential that could be activated under a specific dynamic condition. Future studies with additional chromatin profiling under various conditions would allow for a deeper understanding of the regulatory promoters in B compartments. We now discuss this potential biological interpretation in the revised Discussion (page 24). We also revised the title of Supplementary Table 10 (previously Supplementary Table 9) to "Enrichment of cell-type level primary lead *cis*-eQTL variants in *cis* regulatory elements and epigenomic features in the active (A) and inactive (B) chromatin compartments".

Comment #3. Figures 3 and 4 reference Supplementary Table 10 for heritability and colocalization results, but these are actually in ST11–13. Please correct these references.

Response #3. We thank the Reviewer for catching this typo. We referenced Supplementary Table 12 (previously Supplementary Table 10) for the information on trait abbreviations and data sources. We have now revised these figure legends to reference Supplementary Tables 12 and 14 for Figure 3 and Supplementary Tables 10 and 15 for Figure 4.

Reviewer #2 (Remarks to the Author)

I co-reviewed this manuscript with one of the reviewers who provided the listed reports. This is part of the Nature Communications initiative to facilitate training in peer review and to provide appropriate recognition for Early Career Researchers who co-review manuscripts.

Responses to Reviewer #3
We thank the Reviewer for the helpful comments and suggestions on the manuscript. We have carefully revised the manuscript to address all of the Reviewer's comments. On behalf of the authors, we believe that the manuscript has been substantially improved as a result of these insightful comments. The numbering of the revised pages we provide below refers to the tracked changes version of the revised manuscript. We also provide a clean manuscript. In our responses, we refer to previous papers using their PubMed IDs.

Reviewer #3 (Remarks to the Author):

The manuscript titled "Adipose single cell epigenome and transcriptome localize genetic risk for cardiometabolic disease and accelerated aging" presents an impressive and timely study that tackles the complexity of obesity-associated diseases by dissecting the subcutaneous adipose tissue (SAT) at single-cell resolution.
The integration of snRNA-seq with snm3C-seq to map the 3D epigenomic landscape is particularly commendable. The authors' finding that the active "A" compartments in adipocytes serve as a hub for disease heritability provides a compelling structural mechanism for how genetic risk is translated into phenotype. Furthermore, the identification of cell-type-specific cis-eQTLs that were missed by previous bulk studies highlights the unique value of the dataset. Overall, the experimental design is rigorous, and the manuscript is well-written. A few areas have been identified where additional computational checks and clarifications could further strengthen the authors' conclusions and broaden the impact of the work.

Response to the Reviewer's general comment:
We thank the Reviewer for the helpful and constructive comments. We have now carefully revised the manuscript to address all of the Reviewer's comments. These include multiple additional computational analyses detailed below and revising all aspects that required clarification. As suggested by the Reviewer, we have also now performed the down-sampling analysis to validate the predominance of adipocyte eGenes. Overall, we are very grateful for the Reviewer's thorough feedback as we feel that these revisions have substantially strengthened the conclusions from our work.

Major Comments
Comment #1. Validating the Predominance of Adipocyte eGenes: A key finding is that adipocytes harbor the vast majority of detected eGenes (1,215 out of 1,684). While this aligns with the biological role of adipocytes in SAT, Figure 1 indicates a correlation between the number of nuclei sequenced and the number of eGenes found. Although the authors utilized a modified Z-score to correct for this, a direct empirical validation would be very persuasive.
Suggestion: To definitively rule out sample size bias, it is recommended that the authors perform a down-sampling analysis. The authors should randomly subsample the adipocyte nuclei to match the counts of less abundant cell types (e.g., endothelial or lymphoid cells) and re-run the eQTL mapping. If adipocytes still show a higher number (or higher density) of eGenes compared to other cell types at the same sequencing depth, this will strongly solidify the conclusion that adipocytes are the primary regulatory hub.

Response #1. We thank the Reviewer for this insightful suggestion. We have now performed the suggested down-sampling analysis, in which we randomly subsampled the adipocyte nuclei to match the counts of endothelial and lymphoid cells for each individual. We then repeated the *cis*-eQTL mapping and discovered 808 and 500 eGenes in adipocytes when down-sampling to the number of endothelial and lymphoid cells, respectively. Thus, down-sampling the adipocytes still resulted in larger numbers of adipocyte eGenes than the 190 endothelial and 96 lymphoid cell eGenes we had identified. In line with our previously shown modified Z-score test (Figure 1e), the greater number of eGenes discovered in the down-sampled adipocytes solidify the conclusion that adipocytes are the primary regulatory hub. We have now added these down-sampling results into the revised Results (page 6) and Methods (page 32).

Comment #2. Generalizability of the 3D Epigenome Map: The snm3C-seq data provides a unique view of the chromatin architecture. As noted, this data is derived from 5 female donors. While this is a standard sample size for such a high-complexity assay, discussing its representativeness would be beneficial for the reader.
The authors are advised to add a discussion paragraph or a supplementary analysis addressing the generalizability of the A/B compartment maps. If feasible, comparing the compartment calls with publicly available bulk Hi-C data from human adipose tissue would serve as an excellent "sanity check" to demonstrate that the broad structural features are consistent with the larger population. This would reassure readers that the N=5 dataset is a reliable reference for the broader N=127 cohort.

Response #2. We thank the Reviewer for this helpful comment. We acknowledge the limited sample size and lack of male donors in the reference single cell level snm3C-seq data due to the high-complexity of the assay used, as mentioned by the Reviewer. To the best of our knowledge, there are no subcutaneous adipose tissue bulk Hi-C datasets even with the same sample size available to do an in-depth comparison of the A/B compartment mapping. Furthermore, we note that differences in resolution between single nucleus and bulk tissue data would likely affect these comparisons, which may limit the assessment of the A/B compartment mapping generalizability. We would like to highlight, however, that a substantial proportion of our snRNA-seq data are from females (64%), thus the A/B compartment maps from the cell-type level snm3C-seq data remain relevant and informative for the broad epigenomic localization of the cell-type level *cis*-eQTLs. We have now discussed the limitations of the representativeness of the A/B compartment maps in the limitations paragraph of the revised Discussion (pages 26 and 27).

Comment #3. Robustness of the T2D Prediction Model: The Elastic Net model predicting T2D status using 41 colocalized eGenes is a promising clinical application (AUC 0.74). Given the cohort size (N=127), a single 70:30 train/test split can sometimes be sensitive to how the data is partitioned.
To demonstrate the stability of the model, the authors should consider using a Leave-One-Out Cross-Validation (LOOCV) or a repeated k-fold cross-validation approach (e.g., 50 or 100 iterations). Reporting the mean AUC and its standard deviation from these iterations will provide a more robust estimate of the model's performance and protect against potential overfitting.

Response #3. We thank the Reviewer for this very helpful comment. As suggested, we repeated the random splitting of 70/30 train/test sets with 5-fold inner-CV for 100 iterations to evaluate the robustness of the T2D prediction model. After 100 iterations, we observed a mean AUC of 0.640 (SD=0.062, min=0.557, max=0.807), yielding a moderate predictive power. As our previously reported AUC was 0.74, the model exhibited substantial variability across random splits, likely reflecting the limited cohort size available. We therefore conclude that this analysis requires additional investigation in future, larger snRNA-seq cohorts. To avoid distracting from our main cell-type level *cis*-eQTL and colocalization results, we chose to remove the expression-based prediction analysis from the manuscript.

Comment #4. Clarification of Interaction Analysis (Figure 6b): The finding that the partitioned PRS interacts with BMI to increase T2D risk is highly significant. However, it is not immediately clear in the text whether this specific interaction analysis was powered by the local bariatric cohort (N=127) or if it was conducted using the UK Biobank dataset.
The authors should clarify the data source for this specific analysis in the main text and figure caption. If it was performed in the local cohort, a brief mention of the statistical power or a caveat regarding sample size for interaction terms would be appropriate to contextualize the findings.

Response #4. We thank the Reviewer for this helpful comment. The partitioned PRS interaction analysis was performed using the UK Biobank dataset to maximize statistical power and ensure robust results. However, in response to Reviewer #1's comment #9, we have now tested for the association between the partitioned T2D PRS and BMI. We observed a significant positive association (beta=0.02, *p*-value=5.35×10^{-45}), and thus the partitioned T2D PRS predicts both T2D and BMI. This result also suggests that the observed interaction could reflect the underlying correlation between the T2D PRS and BMI. To further dissect these results, we first residualized BMI on the partitioned T2D PRS and covariates to remove the component of BMI that is driven by the T2D PRS. When we then retested for the interaction between this residualized BMI and the partitioned T2D PRS, we observed that the interaction effect is not significant anymore (*p*-value>0.05) while the main effect of the T2D PRS is significant (beta=0.13, *p*-value=1.36×10^{-40}). This supports the conclusion that the originally observed interaction effect between the partitioned T2D PRS and BMI may be largely due to the underlying association between the partitioned T2D PRS and BMI. To corroborate the genetic effect on T2D at the colocalized loci, we also reexamine the main effect of the partitioned T2D PRS on T2D after accounting for BMI. <u>We discovered that the partitioned T2D PRS is still a significant predictor of T2D (beta=0.11, *p*-value=6.10×10^{-44}), suggesting that the polygenic effects on T2D are not fully explained by BMI.</u> Overall, these results demonstrate complex genetic correlations between the partitioned T2D PRS and both T2D and BMI and reveal polygenic correlations, where the underlying functions and pathways of the colocalized T2D genes also likely have pleiotropic effects on obesity. <u>These new results are in line with the fact that the colocalized loci were discovered using eQTLs from individuals with obesity and that obesity is a main risk factor for T2D</u>. Accordingly, we have now revised the Results (page 20) and Methods (page 43 and 44) to show these partitioned polygenic correlations while also removing the interaction result.

Minor Comments

Comment #1. Methodological Detail: The methods section for snm3C-seq is quite concise. Expanding the Supplementary Methods to include more details on library preparation, sequencing depth per nucleus, and QC metrics would greatly assist the community in reproducing this cutting-edge technique.

Response #1. We thank the Reviewer for this constructive suggestion. We have now revised the Supplementary Methods (page 5–8) to provide a more detailed description of the snm3C seq data.

Comment #2. Terminology: The terms "sn-eQTL" (single-nucleus) and "sc-eQTL" (single-cell) appear occasionally. For consistency, it is recommended that the authors stick to one term (likely "sn-eQTL" given the methods) throughout the manuscript.

Response #2. We thank the Reviewer for the helpful suggestion. For conceptual unity and to improve the searchability of our manuscript, we have now chosen to use “single cell” consistently throughout the manuscript. Accordingly, we now clearly explain in the revised Results (page 5) that we use the term “single cell” for the “single nucleus” RNA-seq sequencing data.

Comment #3. Data Availability: The authors should ensure that the summary statistics for the cell-type-specific eQTLs are made easily accessible (e.g., via a repository or supplementary table). This will be a valuable resource for future meta-analyses.

Response #3. We thank the Reviewer for this constructive comment. We have now deposited the full summary statistics of the *cis*-eQTLs generated from tensorQTL for each cell-type on Zenodo at https://zenodo.org/records/18880304 and will publicly open the access to the data upon acceptance of the manuscript. Additionally, in response to Reviewer #1’s comment #5, we have now performed cell-type level *cis*-eQTL mapping using SAIGE-QTL (PMID: 38798318) and provide the full summary statistics on Zenodo. We have also revised the Data availability statement accordingly (page 45). The data deposited on Zenodo can be accessed by the reviewers using the following private link:
https://zenodo.org/records/18880304?preview=1&token=eyJhbGciOiJIUzUxMiJ9.eyJpZCI6IjYxOTA2YTJkLTY4YzEtNGI1YS1hYmFkLWJjN2UxMWM4NzlkOCIsImRhdGEiOnt9LCJyYW5kb20iOiIzZDcyZDg2ZDViYzkxODg4NDQ5OGM0ZjZlNjQ1YTc3MiJ9.2IUgNYKuCMxC_P5Dc6w21z1xG_UQ1uZ8uIBeMpBEOxs6Ur5SH95sJMtT9iBWor5FyD4ilo9jVQo-TFlsfaZOXQ

Comment #4. Figure 3 and Supplementary Figure 2 appear to visualize the same heritability enrichment results, but the x-axis scales differ noticeably between the two figures. This makes it difficult to directly compare effect magnitudes across the main and supplementary panels. For clarity and consistency, it would be helpful to harmonize the axis ranges or explain the rationale for using different scales.

Response #4. We thank the Reviewer for this helpful suggestion. We have now revised Supplementary Figure 2 to match the x-axis scale to that of Figure 3 for easy comparison. We also provide the full partitioned heritability results in Supplementary Table 14.

Comment #5. The manuscript and Figure 5a reports that 90% of colocalized eGenes are cell type–specific, while the mashr analysis shows that only 30–56% of the eGenes are cell type–specific. I understood these figures reflect different levels of specificity—variant-level colocalization versus gene-level eQTL sharing— but this may be confusing to readers. Clarifying the distinction between these two concepts would improve interpretability and coherence of the results.

Response #5. We thank the Reviewer for this thoughtful comment. As mentioned by the Reviewer, Figure 5a shows the two concepts of cell-type-specificity. Our main goal with this figure and corresponding result was to highlight that not all cell-type-specific colocalizations are observed with cell-type-specific eGenes and that distinct regulatory variants linked to disease risk can act on the same gene in different cell-types. We have now clarified the distinction between the two concepts of cell-type-specificity and better articulated the main interpretation in the revised Results (page 16).

Responses to Reviewer #4
We thank the Reviewer for the helpful comments and suggestions on the manuscript. We have carefully revised the manuscript to address all of the Reviewer's comments. On behalf of the authors, we believe that the manuscript has been substantially improved as a result of these insightful comments. The numbering of the revised pages we provide below refers to the tracked changes version of the revised manuscript. We also provide a clean manuscript. In our responses, we refer to previous papers using their PubMed IDs.

Reviewer #4 (Remarks to the Author)
This manuscript by Lee et al. integrates single-nucleus RNA-seq and single-cell epigenomic data from subcutaneous adipose tissue (SAT) of individuals with obesity to map cell-type-specific cis-eQTLs and link them to cardiometabolic disease (CMD) and aging traits. The authors identify 1,684 eGenes, discover 682 colocalized eQTL-GWAS pairs, and show enrichment of colocalized variants in active chromatin compartments. They also demonstrate predictive utility for T2D using gene expression and a partitioned polygenic risk score (PRS). While the study is technically rigorous, the relatively small sample size (n=127) limits detection of eQTLs in rarer cell types and the obesity-only context may to some extent limit generalizability. Some additional analyses could further increase the impact and resource value of this work.

Response to the Reviewer's general comment:
We thank the Reviewer for the helpful and constructive comments. We have now carefully revised the manuscript to address all of the Reviewer's comments. These include multiple additional computational analyses detailed below and clear articulation of the new findings and the broader impact of our study beyond previous bulk studies. To increase the resource value of this work, we have also now compared our SAT cell-type level *cis*-eQTLs with eQTLs of the two adipose depots from GTEx to assess tissue specificity, as detailed in our response to the Reviewer's comment #4. Furthermore, in response to Reviewer #1's comment #5, we have additionally performed *cis*-eQTL mapping using SAIGE-QTL (PMID: 38798318), a single cell eQTL tool. We have compared the *cis*-eQTL results from the two methodological approaches (pseudobulk vs single cell) and provide the full *cis*-eQTL summary results from SAIGE-QTL as an additional resource for the scientific community. Overall, we are very grateful for the Reviewer's thorough feedback as we feel that these revisions have substantially clarified the main impact and resource value of this work.

Major comments:

Comment #1. The manuscript should better articulate what is fundamentally new beyond previous SAT bulk eQTL studies (e.g., Brotman et al., Nat Genet 2025).

Response #1. We thank the Reviewer for this helpful comment. As suggested by the Reviewer, we have revised Discussion (pages 23 and 24) to better articulate which findings from this study are fundamentally new beyond previous SAT bulk eQTL and colocalization studies (e.g., Brotman et al., Nat Genet 2025=PMID: 39747594) as follows:

First, our study moves beyond previous SAT bulk eQTL and colocalization studies (PMIDs: 28257690, 31564431, 39747594) by using SAT single nucleus RNA-seq data both to discover previously unknown cell-type level *cis*-eQTLs and unravel the cell-type of action for many of the known bulk SAT eQTLs. Using then these cell-type level *cis*-eQTL data in colocalization analyses, we identified 208 new colocalized signals with cardiometabolic diseases that were not previously reported in the SAT bulk colocalization studies. We also resolved the underlying cell-type of action for many of the known bulk SAT *cis*-eQTLs and previously known colocalizations.

Second, our study investigated individuals with obesity unlike the previous studies that comprised predominantly healthy individuals (PMIDs: 28257690, 31564431, 39747594). Thus, we capture obesity-relevant regulatory variants and their links to cardiometabolic disease (CMD) and accelerated aging. Given that increasing number of studies are showing that many *cis*-eQTLs are context-dependent (PMID: 32912663), it is important to investigate groups of individuals with a specific disease or context that have not been included in previous studies to discover additional regulatory variants.

Third, we performed colocalization analysis with eight additional traits that were not previously tested in the SAT bulk colocalization analysis (PMID: 39747594), including two aging (accelerated aging and biological aging), two hematological (monocyte counts and percentage), two inflammatory (c-reactive protein and insulin-like growth factor-1), and two hepatic (alanine transaminase and metabolic dysfunction-associated steatotic liver disease) traits. This is important because these eight traits are known to associate with obesity and related CMDs (PMIDs: 38783156, 20701600, 31440251, 36927772). With these additional eight traits, we discovered 40 new colocalized eQTL-GWAS pairs across the five SAT cell-types, providing novel, more comprehensive insight into the regulatory mechanisms underlying CMDs and accelerated aging.

Fourth, our study extends beyond previous adipose bulk tissue cell-type level eQTL studies (PMIDs: 28257690, 31564431, 39747594) by integrating human cell-type level DNA methylation and three-dimensional (3D) genome organization data to improve localization of CMD variants by uncovering their epigenomic regulatory architecture underlying disease regulatory mechanisms. We utilized our SAT single-nucleus methyl-3C sequencing (snm3C-seq) data with matching cell-types (PMID: 40835891) to characterize the identified cell-type level *cis*-eQTL variants. We show that *cis* regional variants of eGenes are preferentially localized in the transcriptionally active (A) chromatin compartments and enriched for *cis* regulatory elements and epigenomic features. Furthermore, we found that these cell-type level A compartments are significantly enriched for 1) CMD and accelerated biological aging trait heritability and 2) *cis*-eQTL variants colocalized with the trait-associated risk variants, especially in adipocytes. To the best of our knowledge, these types of connections between cell-type level genetic and epigenetic regulation of SAT genes and genetic susceptibility to CMDs and accelerated biological aging have not been identified in humans prior to our study. Thus, our study provides further insights into colocalized CMD and accelerated aging variants by characterizing their cell-type level 3D chromatin architecture. We have now clarified these new results of our study in the revised Discussion (pages 22 and 23).

Comment #2. It is unclear whether sex was included as a covariate in all analyses. According to SAGER guidelines, please clarify and justify why sex-stratified analyses were not feasible.

Response #2. We thank the Reviewer for this insightful comment. In the *cis*-eQTL analysis, we included top expression principal components (PCs) as covariates. Sex was correlated with these expression PCs, suggesting that the effects of sex on gene expression were captured by the expression PCs. Thus, to prevent redundant correction of the data, sex was not explicitly included as a covariate. For the T2D prediction analysis using elastic net regularization and the partitioned polygenic risk score analysis, sex was included as a covariate. We have now clarified these corrections and correlations in the revised Methods (page 31) and added a new Supplementary Figure 6 showing correlations between expression PCs and technical and biological covariates for each cell-type.

We did not perform sex-stratified analyses due to the limited sample size of males (n=46), which would have reduced statistical power to generate robust results. Furthermore, sex-stratified GWAS summary statistics for the partitioned heritability and colocalization analyses were not available for all traits included in this study. Thus, we focused on the datasets that included both sexes and adjusted for sex to maximize power and comprehensively test larger numbers of CMD traits. We have now included the justification for not performing sex-stratified analyses in the revised Discussion (page 27).

Comment #3. The current approach (coloc) to identify shared causal variants between cell-type-specific cis-eQTLs and GWAS signals assumes a single causal variant per locus, which may oversimplify complex loci. To strengthen confidence in the colocalization analyses, key findings should be validated using an alternative method that accommodates multiple causal variants.

Response #3. We thank the Reviewer for raising this important point. We acknowledge the single-causal-variant assumption of coloc. To address this assumption, we performed stepwise conditional analysis to identify conditionally independent signals at each locus in the same way as in the previous SAT bulk tissue colocalization study (PMID: 39747594). Then, we conducted colocalization analysis separately for each conditionally independent signal to isolate individual signals prior to colocalization and thereby accounting the effect of multiple causal variants within a locus. We chose to adopt this two-step conditional and colocalization analysis framework as this framework was used in this large previous adipose meta-analysis (PMID: 39747594), to which we directly compared our results, ensuring methodological consistency and comparability. This framework has also been widely used in literature for complex traits (PMIDs: 28257690, 31564431, 39747594, 35729600, 39173627). Thus, we believe that our approach is robust, generating results that can directly be compared to the key results from previous studies (e.g., PMID: 39747594), while acknowledging the single-causal-variant assumption of coloc in the limitations paragraph of the revised Discussion (page 27).

Comment #4. To increase the ressource value of the manuscript, the authors might compare SAT cell-type-specific eQTLs with visceral adipose tissue (VAT) or GTEx adipose depots to assess tissue specificity.

Response #4. We thank the Reviewer for this helpful suggestion. As, to the best of our knowledge, no VAT cell-type level *cis*-eQTL data are publicly available yet, we performed the comparison analysis of the 1,324 SAT top cell-type-specific eQTLs with the SAT (n=479) and VAT (n=393) bulk *cis*-eQTL results from GTEx (V8, European subset). Focusing on our 1,096 SAT cell-type-specific variants that were tested in both SAT and VAT bulk tissue analysis of

GTEx, 329 (30%) of them were bulk SAT *cis*-eQTLs (p-value<1×10^{-6}) with a concordant direction of effect while 250 (23%) were also bulk VAT *cis*-eQTLs (p-value<1×10^{-6}) with a concordant direction of effect. This significantly greater number of SAT cell-type-specific *cis*-eQTLs discovered in the SAT than VAT bulk tissue *cis*-eQTL analysis of GTEx (p.adj=1.11×10^{-14} by one-sided McNemar's test) supports the conclusion that the SAT cell-type-specific eQTLs are in part tissue-specific.

Additionally, we performed the above comparison analyses for cell-type level lead *cis*-eQTL variants in each of the five SAT cell-types. Of our variants that were also tested in both SAT and VAT bulk tissue *cis*-eQTL analysis of GTEx, we observed that 37% vs 30% in adipocytes, 52% vs 41% in ASPCs, 56% vs 48% in endothelial cells, 30% vs 29% in lymphoid cells, and 43% vs 37% in myeloid cells were also *cis*-eQTLs in the SAT vs VAT bulk tissue *cis*-eQTL analysis of GTEx with a concordant direction of effect, respectively. In line with the SAT cell-type-specific eQTLs, a greater number of cell-type level *cis*-eQTL variants were discovered in the SAT than VAT bulk tissue *cis*-eQTL analysis of GTEx for all cell-types (p.adj<0.05) except for lymphoid cells, further supporting the tissue-specificity. We have now included these comparisons between SAT and VAT to the revised Result (page 12), Methods (page 37), and Supplementary Table 11. Additionally, to unify the metrics provided for the replication analyses, we have revised the Results (page 6) to only provide the number and proportion of the primary lead *cis*-eQTL variants that were also *cis*-eQTLs in the SAT bulk meta-analysis (p-value<1×10^{-6}) with and without considering the concordance of effect direction.

Comment #5. The statement on code availability is insufficient for reproducibility. While the authors indicate that 'no custom code was used', the complexity of the analyses suggests that custom scripts or workflows were necessary. Please provide access to all analysis scripts, pipelines, and parameter settings used in this study via a public repository, with initial access for reviewers. This is essential for assessing the reproducibility of the study.

Response #5. We thank the Reviewer for this comment. We have now deposited the code used in this work on GitHub, and it is available at https://github.com/seunghyuktlee/SAT_snRNA_eQTL_2026. This link to the code is now included in the revised Code availability statement (page 46).
